# Pharmacogenetic Study of Trabectedin-Induced Severe Hepatotoxicity in Patients with Advanced Soft Tissue Sarcoma

**DOI:** 10.3390/cancers12123647

**Published:** 2020-12-04

**Authors:** Maud Maillard, Christine Chevreau, Félicien Le Louedec, Manon Cassou, Caroline Delmas, Laure Gourdain, Jean-Yves Blay, Didier Cupissol, Emmanuelle Bompas, Antoine Italiano, Nicolas Isambert, Corinne Delcambre-Lair, Nicolas Penel, François Bertucci, Cécile Guillemet, Julien Plenecassagnes, Stéphanie Foulon, Étienne Chatelut, Axel Le Cesne, Fabienne Thomas

**Affiliations:** 1 Centre de Recherches en Cancérologie de Toulouse (CRCT), Inserm UMR1037, 31059 Toulouse, France; maillard.maud@iuct-oncopole.fr (M.M.); lelouedec.felicien@iuct-oncopole.fr (F.L.L.); Delmas.Caroline@iuct-oncopole.fr (C.D.); gourdain.laure@iuct-oncopole.fr (L.G.); Chatelut.Etienne@iuct-oncopole.fr (É.C.); 2 Université Paul Sabatier—Toulouse III, 31400 Toulouse, France; 3 Institut Claudius Regaud, Institut Universitaire du Cancer (IUCT)—Oncopole, 31059 Toulouse, France; chevreau.christine@iuct-oncopole.fr (C.C.); manon.cassou.g@gmail.com (M.C.); Plenecassagnes.Julien@iuct-oncopole.fr (J.P.); 4 Medical Oncology Department, Centre Léon Bérard, 69008 Lyon, France; jean-yves.blay@lyon.unicancer.fr; 5 Medical Oncology Department, Institut Régional du Cancer Val d’Aurelle, 34090 Montpellier, France; Didier.Cupissol@icm.unicancer.fr; 6 Medical Oncology Department, Institut de Cancérologie de l’Ouest, 44800 Saint-Herblain, France; Emmanuelle.Bompas@ico.unicancer.fr; 7 Medical Oncology Department, Institut Bergonié, 33000 Bordeaux, France; a.italiano@bordeaux.unicancer.fr; 8 Medical Oncology Department, Centre Georges François Leclerc, 21000 Dijon, France; nicolas.isambert@chu-poitiers.fr; 9 Medical Oncology Department, Centre Francois Baclesse, 14000 Caen, France; c.delcambre@baclesse.fr; 10 Medical Oncology Department, Centre Oscar Lambret—Université de Lille, 59000 Lille, France; n-penel@o-lambret.fr; 11 Medical Oncology Department, Institut Paoli-Calmettes, 13009 Marseille, France; bertuccif@ipc.unicancer.fr; 12 Medical Oncology Department, Centre Henri Becquerel, 76038 Rouen, France; cecile.guillemet@chb.unicancer.fr; 13 Department of Biostatistics and Epidemiology, Gustave Roussy, University Paris-Saclay, 94805 Villejuif, France; STEPHANIE.FOULON@gustaveroussy.fr; 14 Oncostat U1018, Inserm, University Paris-Saclay, Labeled Ligue Contre le Cancer, 94805 Villejuif, France; 15 Medical Oncology Department, Gustave Roussy, 94805 Villejuif, France; axel.lecesne@gustaveroussy.fr

**Keywords:** ABC transporters, advanced soft tissue sarcoma, CYP450, hepatotoxicity, next-generation sequencing, pharmacogenetic, trabectedin

## Abstract

**Simple Summary:**

Trabectedin is a cytotoxic drug used for the treatment of advanced soft tissue sarcoma. One of the most frequent side effects is hepatotoxicity, which occurs in nearly 40% of patients. In this pharmacogenetic study, we aimed to identify genetic polymorphisms that could impair the functionality of liver proteins—as metabolic enzymes or membrane transporters—involved in the production and the elimination of trabectedin and/or its metabolites from hepatocytes. In a prospective cohort of 63 patients, we showed that some variants of P-gp and MRP2 transporters and the well-known CYP3A5*3 variant were associated with hepatotoxicity. With these findings, we provide new biomarkers that might be useful to prevent the risk of hepatotoxicity in patients treated with trabectedin. However, this study is limited by the low number of patients included and should be validated on larger cohorts before any clinical application.

**Abstract:**

Hepatotoxicity is an important concern for nearly 40% of the patients treated with trabectedin for advanced soft tissue sarcoma (ASTS). The mechanisms underlying these liver damages have not yet been elucidated but they have been suggested to be related to the production of reactive metabolites. The aim of this pharmacogenetic study was to identify genetic variants of pharmacokinetic genes such as CYP450 and ABC drug transporters that could impair the trabectedin metabolism in hepatocytes. Sixty-three patients with ASTS from the TSAR clinical trial (NCT02672527) were genotyped by next-generation sequencing for 11 genes, and genotype–toxicity association analyses were performed with R package SNPassoc. Among the results, *ABCC2* c.1249A allele (rs2273697) and *ABCG2* intron variant c.-15994T (rs7699188) were associated with an increased risk of severe cytolysis, whereas *ABCC2* c.3563A allele had a protective effect, as well as *ABCB1* variants rs2032582 and rs1128503 (*p*-value < 0.05). Furthermore, CYP3A5*1 rs776746 (c.6986A > G) increased the risk of severe overall hepatotoxicity (*p* = 0.012, odds ratio (OR) = 5.75), suggesting the implication of metabolites in the hepatotoxicity. However, these results did not remain significant after multiple analysis correction. These findings need to be validated on larger cohorts of patients, with mechanistic studies potentially being able to validate the functional consequences of these variants.

## 1. Introduction

Trabectedin (Yondelis, PharmaMar, Madrid, Spain) is a synthetic alkaloid drug originally isolated from the Caribbean ascidian *Ecteinascidia turbinata*. This multitarget antineoplastic agent has the ability to alkylate the exocyclic nitrogen-2-position of guanines of the DNA’s minor groove and to disrupt the interactions between nuclear proteins and DNA [1]. Trabectedin can also interact with tumor microenvironment and repair processes [2], making it a complex molecule with a unique spectrum of cytotoxicity on various cell lines including sarcomas [3,4]. In clinical practice for the treatment of advanced soft tissue sarcoma (ASTS), Yondelis is given after a first-line chemotherapy composed of anthracyclines or ifosfamide, or if patient presents a contraindication to their use [5,6].

Soft tissue sarcomas (STSs) are a group of rare and heterogeneous malignancies. For trabectedin, pharmacogenomic biomarkers have been previously described such as germline and somatic variants of *BRCA1/2* or repair process-associated genes encoding for excision repair cross-complementation group 1 (ERCC1) and 5 (ERCC5) [7]. A haplotype of *BRCA1* has been associated with better treatment outcomes in terms of progression-free survival (PFS) and overall survival (OS) [8]. Moreover, patients with high expression of the functional proteins ERCC1/5 may have an improved PFS and OS compared to low expressers, being even more so if *BRCA1* or *BRCA2* are lacking [9,10].

Trabectedin is extensively metabolized in the liver into several compounds that are further eliminated mainly in the urine and feces [11,12,13]. Mass balance studies showed that the eliminated part predominantly consisted in metabolites principally eliminated in feces and that less than 1% of the unchanged form of trabectedin is retrieved both in urine and feces, confirming the high contribution of hepatic metabolism in its elimination [14].

The main adverse effects of trabectedin reported in phase 1 clinical trials were myelosuppression; liver toxicity; nausea; emesis; asthenia; and, more rarely, severe rhabdomyolysis [15,16]. In these trials, 30–40% of the patients experienced liver toxicity, mainly consisting of serious (grade ≥ 3 of the National Cancer Institute’s Common Terminology Criteria for Adverse Events, NCI-CTCAE) but transient elevation of transaminases (aspartate aminotransferases (AST) and alanine aminotransferases (ALT)). Because of the reversibility of the increase of the transaminases, biochemical hepatic toxicity was not considered dose-limiting [17], but instead considered schedule-dependent [18]. Subsequently, other liver abnormalities have been reported in patients, such as elevation of bilirubin, alkaline phosphatases (AP), and gamma-glutamyl transferases (γ-GT), confirming that hepatotoxicity remains the main adverse event of trabectedin [19].

The first observations of hepatic damages related to trabectedin were made during preclinical studies on rodents [20,21]. However, it was unclear as to whether they were related to the production of toxic metabolites or the drug itself. Reid et al. [22] showed that trabectedin is degraded in human microsomes by phase I enzymes from the cytochrome P450 (CYP450) family, mainly by CYP3A4, and also by CYP3A5, 2C9, 2C19, 2D6, and 2E1 but in lesser extent [23]. To date, no study has clearly identified the major metabolites involved in toxicity, mostly because of their very low concentrations in plasma associated with a lack of sensitivity of the detection methods [11,12]. Elimination pathways involving ATP-binding cassette (ABC) transporters were also widely studied in order to understand if the accumulation of trabectedin and/or its metabolites may be responsible for hepatic damages. In preclinical studies, hepatic toxicity was increased in *Abcb1*/P-glycoprotein (P-gp), *Abcc2/*Mrp2, or *Abcc3*/Mrp3 knock-out mice [24,25]. Importantly, this toxic effect was worsened when CYP3A enzymes were overexpressed [24], reinforcing the hypothesis of toxic metabolites involved in trabectedin hepatotoxicity.

Adversely, Brandon et al. [26] showed in an in vitro study that HepG2 cells treated with CYP-derived metabolites were less affected than by trabectedin itself. Furthermore, the use of a high dose of dexamethasone in clinical care, a potent CYP3A4 and P-gp inductor, decreases the risk of toxicity when administered 24 h before trabectedin without compromising its efficacy [27]. Furthermore, Lee et al. [28] demonstrated that the biliary excretion of trabectedin was impaired in *Abcc2*/Mrp2-deficient rat, and that the induction of *Abcc4*/Mrp4 by L-buthionine-sulfoximine decreased the cytotoxicity even with high-concentrations of trabectedin. These mechanisms of trabectedin disposition in hepatocyte are strongly dependent on the integrity of these hepatic proteins, and nonfunctional enzymes or transporters due to genetic variants could lead to an increased toxicity.

In 2013, Laurenty et al. [29] presented a case of lethal hepatotoxicity in a patient treated with trabectedin. After only two cycles, the patient’s transaminases increased six times over the upper normal limit (UNL), with no normalization after stopping the treatment. The patient died of this major and irreversible liver toxicity one year later. The pharmacogenetic exploration of the patient’s DNA revealed that he carried some deleterious genetic variants of *ABCC2*/MRP2 and *ABCB1*/Pg-p. Three years later, Rathinamanickam et al. [30] reported a second case of irreversible but non-lethal hepatotoxicity. Unfortunately, no pharmacogenetic investigation was performed.

On the basis of these case-reports, and the fact that the role of pharmacogenetics on trabectedin-related toxicity was poorly documented, we hypothesized that genetic variations of hepatic transporters of hepatic transporters and metabolism enzymes may influence the disposition of trabectedin and consequently increase the risk of liver toxicity. Thus, this ancillary pharmacogenetic study of the TSAR clinical trial (NCT02672527, Institut Gustave Roussy, Villejuif, France) was conducted to prospectively investigate the role of the single-nucleotide polymorphisms (SNPs) of CYP450 and ABC drug transporters on the incidence of trabectedin-related severe hepatotoxicity.

## 2. Results

### 2.1. Patients’ Characteristics and Toxicity Data

Table 1 summarized the demographic and clinical characteristics of the 63 patients included in this ancillary pharmacogenetic study. In the TSAR clinical trial, patients were divided in two comparative groups, resulting in 31 patients treated with trabectedin vs. 32 who received best supportive care (BSC). As a cross-over was allowed between the two arms, every patient of the pharmacogenetic cohort was treated with at least one cycle of trabectedin. Treatment was delayed because of hepatotoxicity for six patients (9.5%) and associated with a dose reduction of the following courses for four of the patients. In total, trabectedin dose was reduced for 17 patients (27%). Furthermore, treatment was stopped because of acute hepatotoxicity for two patients of the trabectedin arm that occurred during the two first cycles.

Occurrences of hepatic side effects after cycle 1 and cycle 2 of trabectedin were considered as two classes: severe (grade 3 (G3) and grade 4 (G4)) vs. not severe (grades 0, 1, or 2), as shown in Table 1. Forty-three percent (27/63) of the patients experienced a severe cytolysis during the two first courses of trabectedin at a dose of 1.5 mg/m^2^. For most of them (23/27, 85.2%), this elevation was transient, and AST/ALT normalized after the second cycle of treatment. In the cohort, 4.7% (3/63) patients experienced a severe cholestasis with elevation of γ-GT associated with AP and total plasma bilirubin. Isolated elevation of γ-GT with normal transaminases was reported in seven patients (11.1%). In order to evaluate the impact of SNPs on overall hepatotoxicity, we grouped AST/ALT, γ-GT, total plasma bilirubin, and AP. Thus, a total of 36 out of 63 patients (57.1%) experienced at least one severe hepatotoxic event after two courses of trabectedin.

Since trabectedin is a third-line treatment, each patient had already undergone prior adapted chemotherapy or targeted therapy for their STS. Among these, doxorubicin and dacarbazine were the most likely to induce mild and transient liver abnormalities [31]. In our cohort, 98.4% of the patients received doxorubicin before trabectedin, and 15/63 were previously treated with dacarbazine (Table 1). Ten patients were previously treated with pazopanib, which is commonly associated with transaminase elevation [31]. However, there was no significant association between prior treatment and the occurrence of severe hepatotoxicity (data not shown). Of note, every patient had normal serum values of the biological hepatic parameters before trabectedin initiation.

### 2.2. Evaluation of the NGS Data Quality and Building of the Genetic Database

DNA was successfully extracted from the whole blood samples for 63 patients. Quality was sufficient for library conception and subsequent Next-Generation Sequencing (NGS) analysis. Then, sequencing chips were loaded with pooled libraries at a rate higher than 90%, with the mean of the total reads being near 3 million per chip. As genomic DNA was analyzed, minimal sequencing depth was fixed to 20X and variant with inferior depth was considered as not covered [32]; thereby, mean total sequence read depth reached 500X. Overall, the total mean sequencing coverage of the 276 amplicons was 86.9%. Mean alignment to reference human genome (hg19) was > 99%.

After applying a filter according to the cohort minor allele frequency (MAF) fixed at the threshold of 0.01, we collected a total of 276 genetic variants. Among these, 44 were excluded because of a genotyping rate less than 80%, or a variant frequency of 100% (i.e., monomorphic allele). Fourteen variants were not considered because of deviation from the Hardy–Weinberg equilibrium (HWE) with *p <* 0.05. The final database contained 208 SNPs that were visually checked with the Alamut software. Among them, 206 were referenced in the Single Nucleotide Polymorphism Database (dbSNP) and 2 were unknown. Variants included 203 single-nucleotide variations and 5 deletions. MAFs of collected SNPs were compared to those of the Caucasian population, and no strong deviance was observed.

Among the 47 candidate SNPs selected after a literature review, 39 were correctly genotyped (genotyping rate > 80%), but 3 variants were further eliminated because of deviation from HWE (rs1135840, rs1065852, and rs2740574) and 1 was found to be monomorphic (rs3765534). Finally, 35 SNPs were selected for primary analysis.

### 2.3. Pharmacogenetic Association Studies

#### 2.3.1. Primary Analysis Based on Literature-Selected SNPs

The significant results (on the basis of *p*-value) of the association analysis restricted to the 35 selected SNPs are presented in Table 2. None of the variants remained significant after multiple testing correction (all variants presented a false discovery rate (FDR) > 0.05). However, the variants with the lowest *p*-values and FDR may be considered as interesting leads to explain inter-patient variability regarding hepatotoxicity occurrence. First, the variant T allele of *ABCB1* rs1128503 (c.1236C > T, a synonymous variant, p.Gly412=) was associated with a lower risk of severe AST/ALT elevation. Indeed, the dominant model revealed that only 35% (14/40) of the allele T carriers did experience a severe cytolysis (T/C-T/T vs. C/C, *p* = 0.015, odds ratio (OR) = 0.25, 95% confidence interval = (0.08–0.80)), whereas 68.4% (13/19) of patients carrying the C/C genotype, considered as the most frequent, developed a severe cytolysis during the two first cycles of trabectedin.

Additionally, *ABCB1* missense variant rs2032582 (c.2677G > T, A p.Ala893Thr/Ser) had a protective effect against the risk of overall hepatotoxicity (T/T vs. GG-G/T,A, OR = 0.22 (0.05–0.91), *p* = 0.027), supported by the large amount of homozygous GG patients in the group of toxicity (12/18, 67%) and the fact that the majority of the carriers of two copies of variant allele T did not experience a hepatotoxic event (8/11, 72.7%).

Three variants of the *ABCC2* gene significantly influenced the variation of transaminases. Missense variant rs2273697 (c.1249G > A, p.Val417Ile) was found in 14 cases patients, including 13 heterozygous patients and 1 homozygous patient. Carriers of the A allele showed an increased risk of undergoing a severe ALT/AST elevation (G/A-A/A vs. G/G, OR = 3.63 (1.22–10.83), *p* = 0.018).

The analysis also revealed that carriers of at least one variant allele of rs17222723 (c.3563T > A, p.Val1188Glu) had a significantly decreased risk of severe cytolysis (OR = 0.11 (0.01-0.91), *p* = 0.010). Indeed, severe cytolysis occurred for 51% of the T/T genotyped patients (26/51), whereas only 1 heterozygous patient out of 10 carrying the A allele developed a severe cytolysis. Finally, *ABCC2* synonymous variant rs8187707 (c.4488C > T, p.His1486=) was modestly associated with a protective role against severe cytolysis (C/T vs. C/C, OR = 0.15 (0.02–1.35), *p* = 0.045, but FDR = 0.371). The minor allele of the *ABCG2* rs7699188 intron 2 variant (c.-15994C > T) was significantly associated with an increased risk of severe cytolysis (C/T-T/T vs. C/C, OR = 3.41 (1.07–10.87), *p* = 0.034). Indeed, among the 23 patients experiencing cytolysis, 56.5% (13/23) were carriers of at least one T allele.

Since the incidence of severe cholestasis was limited to three patients, association between genetic variants and cholestasis was not evaluated.

Among the patients, seven presented an isolated severe elevation of γ-GT. Genetic association analysis revealed that *ABCC2* rs2273697 was associated with γ-GT isolated increase with a protective role of the variant allele (G/A-A/A vs. G/G, *p* = 0.044), as no patient carried this allele in the case group. However, the FDR for this variant was high, indicating a risk of misinterpretation of this association.

Finally, every patient treated with one or two cycles of trabectedin and who experienced at least one severe hepatic adverse effect were grouped. Interestingly, the analysis revealed that the wild-type allele A of *CYP3A5* rs776746 (c.6986A > G) coding for the CYP3A5*1 genotype was associated with this overall hepatotoxicity. The incidence of severe hepatic toxicity was higher in heterozygous *1/*3 patients (A/G vs. A/A vs. G/G, *p* = 0.012, OR = 5.75 (1.16-28.55)). Indeed, 85.7% of the heterozygous patients (12/14) had at least one severe elevation of their hepatic enzymes. In the cohort, two patients were homozygous *1/*1 (A/A) but did not experience any hepatotoxic event.

*ABCC4* rs9516519 (c.*3261A > G, 3′ untranslated region (3′UTR) variant) was also associated with severe overall hepatotoxicity, revealing a protective role of the variant allele G (T/G-G/G vs. T/T, OR = 0.31, *p* = 0.048).

These results were confirmed with the cross-validation procedure. The ORs generated with the k-fold procedure (presented in Table S1) remained significant (i.e., strictly higher or lower than 1) and included the ORs reported in the univariate analysis (presented in Table 2), confirming the robustness of the statistical results. Moreover, the 95% CI for AUCs (area under the receiver operating characteristic curve) were superior to 0.5 (see Table S1), which indicated a putative predictive effect for the selected variants.

#### 2.3.2. Exploratory Analysis

In order to expand the analysis to rare and poorly or never described variants, we analyzed the totality of the 208 SNPs revealed by the NGS. The significant results are summarized in Table S2. In addition to the SNPs previously revealed during the primary analysis, 18 SNPs were significantly associated with severe cytolysis, 9 with severe isolated elevation of γ-GT, and 16 with severe overall hepatotoxicity. Those SNPs mainly concerned genes of the ABC transporters families such as *ABCB* (i.e.*, ABCB1*), *ABCC* (i.e.*, ABCC2, C3*, and *C4*), and *ABCG* families (i.e.*, ABCG2*), but also those encoding for CYP2D6 and CYP2E1.

Among the total of 51 significant genotype–toxicity associations, none remained significant after multiple testing with FDR correction. Therefore, only the most significant results (*p <* 0.01), which mainly consisted of ABCC variants, were examined, and are presented in Table 3.

*ABCC2* rs17216282 intron variant (c.4146 + 11G > C) was found to decrease the risk of severe cytolysis (G/C vs. G/G vs. C/C, *p* = 0.009, OR = 0.1 (0.01–0.87)). Similarly, carriers of at least one variant allele of the *ABCC4* rs1751005 (c.1727 + 91G > A) experienced a lower risk of severe cytolysis than wild-type carriers (G/A-A/A vs. G/G, OR = 0.18 (0.05–0.74), *p* = 0.009). On the other hand, frequency of severe cytolysis was higher in patients carrying two copies of the 3′UTR variant of *ABCC4* rs4148553 (c.*694G > A) compared to G/G and G/A patients (*p* = 0.006).

The analysis of the overall hepatotoxicity revealed 18 significant SNPs, including 8 concerning *ABCC4*. Among the most significant ones with a *p*-value < 0.01 and low FDR, rs1751005 variant was revealed and, as previously described in severe cytolysis analysis, carriers of at least one minor allele A were found to be less likely to have a severe overall hepatotoxicity (OR = 0.18 (0.05-0.61), *p* = 0.004). Furthermore, results showed that a single base deletion of *ABCC4* (rs11568647 c.2213 + 108delC) was significantly associated with a decreased risk of overall hepatotoxicity, and no carrier of this deletion presented any hepatic adverse event (C/del vs. C/C, *p* = 0.003). In addition, heterozygous patients for *ABCC3* rs2072365 (c.2714 + 29C > T) had a 5.92-fold higher risk (1.83–19.20) of severe overall hepatotoxicity compared to C/C wild-type patients (*p* = 0.003), as well as the heterozygous patients for *ABCC3* rs4148415 (c.2600-123C > T, C/T vs. C/C vs. T/T, OR = 8.36 (2.26–31), *p* = 0.001).

In the analysis of the patients with isolated elevation of γ-GT, we found six variants of the *CYP2E1* gene (rs2249694, rs2249695, rs2480256, rs2480258, and rs2480259), two variants for *ABCC2* (rs2273697 and rs4148395), one for *ABCC3* (rs72837544), and one for *ABCC4* (rs2274408) to be significant in this analysis; however, the *p*-values were > 0.01 and FDR was high, suggesting that these results have a low predictive value.

As mentioned in the previous analysis, the cross-validation procedure provided confirmation of the consistency of these results, as presented in Table S1.

#### 2.3.3. Haplotype Analysis

According to the results of exploratory analysis, *ABCC2* gene was highly represented in the severe cytolysis group (Table S2); thus, a haplotype analysis was conducted on this gene with significant SNPs.

**Figure 1:**
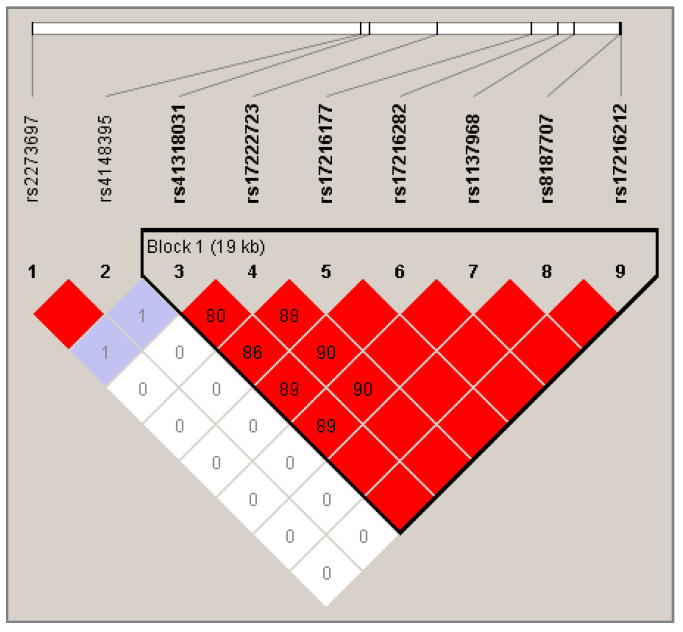
Linkage disequilibrium map of *ABCC2* SNPs. The color of the lozenge represents the |D’| parameter and is filled with R2 value. The redder the lozenge and higher the value, the stronger the linkage between two SNPs. Absence of R2 value within the lozenge means R2 = 100% = complete linkage disequilibrium (LD).

The linkage disequilibrium (LD) map was generated with Haploview and is shown in Figure 1. A block of seven SNPs was highlighted, covering a large region of 19 kb: rs41318031, rs17222723, rs17216177, rs17216282, rs1137968, rs8187707, and rs17216212.

Association between these polymorphisms and severe cytolysis was assessed by performing score tests with a fixed number of SNPs (from seven to two) situated at different positions in the block. Results are shown in Figure S3. According to these results, the most significant regions within block 1 associated with severe cytolysis contained two SNPs—rs1137968 and rs8187707 (*p* = 0.013 for dominant model). Since they are in complete LD (*R^2^* = 100%), haplotype analysis would not bring any advantage over single SNP consideration. In order to evaluate the effect of the association of SNPs included in the haploblock 1 and SNPs outside the haploblock 1, we selected rs8187707 as a tag-SNP using the tagger option of Haploview. We then performed an analysis between rs8187707 and rs2273697. Results showed that AC genotype (i.e., variants allele of rs2273697 associated with wild-type allele of rs8187707) was associated with an increased risk of severe cytolysis in the cohort (frequency 17%, OR = 3.72 (1.15–11.99), *p* = 0.028, additive model).

## 3. Discussion

To our knowledge, this study is the first to assess the association between trabectedin-induced hepatotoxicity and the polymorphisms of some pharmacogenes encoding for CYP450 enzymes or drug transporters in a prospective cohort of patients suffering from ASTS.

In phase 1 clinical studies, transient cytolysis was the main liver abnormality reported [15,17,19], which was confirmed in our cohort with 43% of the patients suffering from severe AST/ALT increase (≥ grade 3, CTCAE) following early cycles of trabectedin.

In our primary analysis, five genetic variants belonging exclusively to the ABC transporter gene family were associated with severe cytolysis, including three variants of *ABCC2* encoding for the multidrug resistance-associated protein 2 (MRP2). First, variant alleles of rs17222723 (c.3563T > A, exon 25, p.Val1188Glu) and rs8187707 (c.4488C > T, exon 31, p.His1486=) were significantly associated with a decreased risk of cytolysis (OR < 0.2, FDR = 0.15 and 0.37, respectively). Functional and clinical studies reported that c.3563A allele was associated with an increased expression and activity of MRP2 both in vitro [33] and in samples collected from patients undergoing liver resection [34]. The subsequent haplotype analysis on *ABCC2* showed that these two variants were in complete LD and belonged to a haploblock of seven SNPs, all associated with a decreased risk of cytolysis in the exploratory analysis. Taken together, these data suggest that the c.3563T > A variant may be responsible for the protective effect observed for the other SNPs of the haploblock (including c.4488C > T) and might be linked with an increased efflux of trabectedin to the bile through MRP2 [35]. Of note, the haplotype association tests did not identify any combination of SNPs that displayed a stronger effect than single locus markers.

On the contrary, *ABCC2* rs2273697 (c.1249G > A, exon 10, p.Val417Ile) was found to increase the risk of severe cytolysis 3.6-fold in patients carrying the A allele (*p* = 0.018). This variant was also found significant in the genetic association of these polymorphisms with the isolated increase of γ-GT (*p* = 0.044). However, the OR was not calculable because no patient carried the variant allele in the case group, and the high FDR may indicate a risk of misinterpretation of this association between variant and increase of these hepatic enzymes, which are not exclusively biomarkers of liver damage [36]. Functional studies showed that this variant was associated with a decrease of the substrate-dependent activity of MRP2 [33], caused by the replacement of a valine in the recognition and binding site of conjugated xenobiotics [37]. Oppositely, Deo et al. [38] observed a significantly higher expression of MRP2 in the liver of c.1249A carriers vs. wild-type livers and hypothesized that this SNP would increase the expression of MRP2 while reducing its activity. In our study, a decreased efflux of trabectedin or its metabolites may be responsible for the higher cytolysis severity in patients carrying the variant 1249A allele, as it has already been demonstrated for carbamazepine, whose accumulation exposed patients to neurological toxicity [39].

Finally, the haplotype analysis performed on the nine SNPs that were significant in the exploratory analysis revealed that seven SNPs belonged to the same haploblock, whereas rs4148395 and rs2273697 were both in LD but outside the block 1. Within the haploblock 1, the haplotype association tests did not identify any combination of SNPs that displayed a stronger effect than single locus markers.

In the present study, the T allele of the well-described *ABCB1* rs1128503 (c.1236T > C, exon 16, p.Gly412=) was associated with a significant reduced risk of severe cytolysis after trabectedin treatment (*p* = 0.015, OR = 0.25 (0.08–0.80)). As a result of a synonymous mutation, the consequences of rs1128503 on the P-gp functionality are controversial [40]. Consistently with some previous studies, we found that this variant provided a protective effect, whether it was present in one or two copies. In a retrospective clinical study, Beuselinck et al. [41] demonstrated that T/T patients needed less dose reduction of sunitinib compared to C/C or C/T individuals. Conversely, Salama et al. [42] showed that this variant was associated with an intracellular accumulation of P-gp substrates because of a decreased efflux function.

Interestingly, *ABCB1* rs2032582 T allele (c.2677G > T, A) was associated with a lower risk of overall hepatotoxicity (*p* = 0.027, OR = 0.22 (0.05-0.91)). However, this controversial variant has previously been described for increasing or decreasing the Pg-p activity, or having no change in drug exposure and drug effects [43]. The ability of P-gp to transport trabectedin has previously been validated in preclinical and clinical studies [24,25,27], and an enhanced biliary efflux could decrease the risk of cytotoxicity in hepatocytes.

Overall, these results highlight the importance of functional biliary transporters, such as P-gp and MRP2, in the elimination of trabectedin, as has been previously suggested [24,28]. Polymorphisms of their encoding genes could be considered as important biomarkers for the prevention of the risk of hepatotoxicity.

By using NGS as a high-throughput tool of sequencing, we reported new intron variants for *ABCC3*/MRP3, including rs2072365 (c.2714 + 29C > T) and rs4148415 (c.2600-123C > T). *ABCC3* rs2072365 strongly increased the risk of overall hepatotoxicity (*p* = 0.003, OR = 5.92 (1.83–19.20), FDR = 0.242), and especially severe cytolysis (*p* = 0.046, OR = 2.99 (1.01–8.84)). In Bai et al.’s [44] recent study, variant allele of rs4148415 was not associated with a risk of anti-tuberculosis drug-induced hepatotoxicity. However, in our study, this variant importantly increased the risk of trabectedin-related liver toxicity (*p* = 0.001, OR = 8.36 (2.26–31), FDR = 0.24). Of note, all the *ABCC3* variants identified in the exploratory analysis were associated with an increased risk of hepatotoxicity, confirming the previously suggested role of MRP3 in the detoxification of trabectedin or related bioproducts from the liver directly in the bloodstream [24,28].

Nine SNPs of *ABCC4*/MRP4 were identified in our exploratory analysis, but most of them were not previously described. Yet, we found that rs9516519 (c.*1372A > C), which leads to the loss of a microRNA (miRNA) binding site in the 3′ untranslated region (3′UTR) [45], was associated with a decreased occurrence of overall hepatotoxicity (*p* = 0.048, OR = 0.31 (0.10–1.01)). Removing the inhibition caused by miRNA may induce a higher expression of MRP4 protein to the basolateral membrane of hepatocytes and decrease the liver toxicity, as previously suggested for MRP3.

In the cohort, 30% of the patients were found to be heterozygous for the *ABCG2* intron 1 promoter variant rs7699188 (c.-15994C > T) among them, 63.2% had a severe increase of their AST/ALT. Genotype toxicity analysis revealed that T allele carriers had a 3.4-fold enhanced risk of toxic cytolysis when treated with trabectedin (*p* = 0.034). Poonkuzhali et al. [46] found that carriers of the rs7699188 T allele had a higher expression of liver BCRP (breast cancer-resistant protein; encoded by *ABCG2*) due to the gain of a transcription factor-binding site. Moreover, the C/T and T/T patients in their clinical cohort had a higher apparent clearance of imatinib compared to C/C individuals, suggesting that its elimination relies on a functional BCRP. On the contrary, De Mattia et al. [47] demonstrated that this variant was significantly associated with grade 3–4 non-hematological toxicity of irinotecan (*p* = 0.0012), suggesting that a gastrointestinal accumulation of its active but toxic metabolite SN38 may result in a local increased toxicity.

Gathering every patient who had experienced a hepatotoxic event in the early cycles of trabectedin allowed us to highlight the well-described variant *3 of *CYP3A5* gene (rs776746 c.6986A > G). In our cohort, heterozygous carriers (*1/*3) had a 5.75-fold higher risk to present a grade 3/4 hepatic event when treated with trabectedin (*p* = 0.012) in comparison with other genotypes.

This splice site acceptor variant was first described by Kuehl et al. [48], who demonstrated that the most frequent G allele (or *3) abolished the activity of the CYP3A5 enzyme by generating a variant exon 3B containing a premature stop codon. Carriers of one or two copies of the ancestral allele *1 are then considered as two categories: moderate metabolizers and high metabolizers, respectively [49]. Therefore, numerous examples of toxicant metabolism involving CYP3A5*1 can be found in the literature, such as acetaminophen-related liver failure caused by the accumulation of its toxic metabolite N-acetyl-p-benzoquinone-imine (NAPQI) [50].

The involvement of a possible activating metabolism through CYP3A5 supports the hypothesis of the formation of toxic metabolites from trabectedin [22]. Van Waterschoot et al. [24] initiated these assertions by noticing that the knock-out of transporters such as P-gp, Mrp2, and Mrp3 in transgenic mice drastically increased the trabectedin hepatic toxicity, whereas it was decreased when Cyp3A was also lacking. These results meant that CYP3A enzymes were involved in the formation of toxic metabolites then eliminated by membrane transporters, as demonstrated by Beumer et al. [25].

Although Lee et al. [28] showed that the biliary excretion of trabectedin occurred through MRP2, the interaction of trabectedin and/or its metabolites with MRP3 and MRP4 remains to be elucidated. However, the same study showed that trabectedin toxicity could be attenuated when Mrp3 and/or Mrp4 expression is upregulated in sandwich-cultured primary rat hepatocytes [28].

Thus, these previous results obtained in vitro or in vivo, taken together with our findings in clinical samples, support the essential role of ABC transporters (and especially MRP2, MRP3, and MRP4) in the detoxification of trabectedin toxic metabolites.

In the clinical case reported by Laurenty et al. [29], the variant *ABCC2* c.-24C > T (rs717620) was hypothesized to be part of the trabectedin-induced liver damage, since the patient was carrying the homozygous variant genotype. However, in the present pharmacogenetic study, this variant was not significantly associated with any hepatic side effects. It should be acknowledged that the pharmacogenetic investigation in the case report only focused on a very limited number of SNPs, contrary to the larger analysis presented here.

The wide variety of the metabolites [13] produced by the numerous enzymes and eliminated by transporters complicates the interpretation of interindividual variability applied to trabectedin metabolism in liver. In addition to this, the complexity of trabectedin mechanism of action and its potential effects on liver remain to be elucidated. Recent findings showed that it may modulate the tumor microenvironment by activating the production and the release of cytokines [51,52]. It is known that deregulation of pro-inflammatory factor production may result in the downregulation of CYP450s and other enzymes involved in drug metabolism [53]. This observation adds a supplementary level of complexity in understanding the mechanism of hepatotoxicity of trabectedin, for which its ability to regulate its own metabolism via a host-mediated reaction may cover up the role of polymorphisms.

The main limitation of our study is the non-significance of these statistical analyses after multiple testing correction (all variants presented as FDR > 0.05). However, the variants discussed above may be considered to be interesting leads representing pharmacogenetic biomarkers associated with a higher risk of hepatotoxicity. In absence of external dataset for validation, the robustness of these significant associations was confirmed with a k-fold cross validation.

Besides its originality, one of the strengths of our study is the use of clinical samples collected within a prospective clinical trial. However, the results are limited by the low number of patients that may induce an over- or underrepresentation of the genetic variants in a small cohort. ASTS are a group of rare tumors, and the use of trabectedin is constantly reconsidered as authorities have argued that it does not bring benefit in terms of global survival compared to dacarbazine treatment or best supportive care [54,55]. However, considering the poor alternatives existing for ASTS, trabectedin is still a part of the therapeutic arsenal, particularly for the treatment of lipo- and leiomyosarcomas [55]. Moreover, trabectedin may also be used for the treatment of recurrent ovarian cancer [56], exposing a large number of patients to the risk of hepatotoxicity [57]. Overall, our results need to be validated in larger independent cohorts, but the potential of these pharmacogenetic biomarkers is high since they could help to identify patients susceptible to undergoing severe hepatotoxicity during the first cycles of treatment.

## 4. Materials and Methods

### 4.1. Patients

This pharmacogenetic study is an ancillary study of the French clinical trial TSAR (NCT02672527, Institut Gustave Roussy, Villejuif, France) [58]. This study was a phase III open-label randomized trial, investigating the benefit of trabectedin vs BSC. A total of 103 patients with ASTS were randomly distributed into two comparative groups, and a cross-over was possible if patients from the BSC arm showed signs of tumor progression (according to the Response Evaluation Criteria in Solid Tumours, RECIST). For both groups, treatment was maintained until tumor progression or non-tolerable toxicity, or according to the patient’s desire to stop the treatment.

At the inclusion for TSAR trial, an EDTA (ethylenediaminetetraacetic acid) whole blood sample was collected for every patient who gave their written informed consent for genotyping. Thus, a total of 63 patients were included in this pharmacogenetic study. Initially, 31 patients (49%) belonged to the trabectedin arm and 32 patients (51%) received BSC. After the cross-over, all the patients were treated with trabectedin at the starting dose of 1.5 mg/m^2^ adjusted with body surface area.

### 4.2. SNP Selection and Genotyping

#### 4.2.1. SNP Selection

A thorough bibliographic research was carried out to select the most relevant genes involved in trabectedin disposition and the candidate SNPs for each gene. Eleven genes were retained: CYP450 genes: *CYP3A4, CYP3A5, CYP2C9, CYP2C19, CYP2D6*, and *CYP2E1*, and ABC transporters: *ABCB1, ABCC2, ABCC3, ABCC4*, and *ABCG2.*

SNPs were collected from international biomedical databases such as Medline (PubMed), PharmaGKB, PharmVar, and the NCBI SNP database (dbSNP). Only SNPs with MAF ≥ 0.01 in the Caucasian population were retained. Thus, a total of 47 variants of CYP450 and ABC transporter families were selected for the primary analysis, which are summarized in Table S4.

As the genotyping method was the use of next-generation sequencing targeting the coding sequences and untranslated regulation regions of the 11 selected genes, we also conducted a larger exploratory analysis using all the variants provided by the NGS in order to explore never or poorly described variants.

#### 4.2.2. Customized Ion Ampliseq Panel for Gene Targeting

An Ion Ampliseq Cancer Hotspot Panel primers panel for NGS was designed with Ion Ampliseq Designer (https://www.ampliseq.com, ThermoFisher Scientific, Carslbad, CA, USA). These couples of primers targeted coding sequences and untranslated regulation regions of 11 genes previously described as involved in trabectedin metabolism and elimination. Additional relevant SNPs described for these genes located outside coding and UTR regions were added in the .*bed* file with a range of 200 base pairs (bp) around the position of the variant.

#### 4.2.3. Libraries Preparation and Amplification for Next-Generation Sequencing

Patient genomic DNA was extracted from whole blood samples with the EZ1 DNA extraction kit for EZ1 Advanced instrument (Qiagen, Chatsworth, CA, USA), and quantified by spectrophotometry with Nanodrop ND-8000 (ThermoFisher Scientific). Library construction was performed using 10 ng of DNA amplified with primers contained in the custom-designed panel mentioned above and the Ion AmpliSeq Library Kit Plus (ThermoFisher Scientific) according to the manufacturer’s protocol. Briefly, after amplification and digestion, amplicons were barcoded and purified in a two-step procedure with Agencourt AMPure XP Reagent (Beckman Coulter, Brea, CA, USA) containing magnetic beads, and eluted in 50 µL of Tris-EDTA (Low-TE) buffer, composed of 10 mM of Tris-HCl (pH 8.0) and 0.1 mM of EDTA. Sizing and quantification of the libraries were evaluated with the Agilent High Sensitivity DNA kit (Agilent technologies, Waldbronn, Germany) for Agilent bioanalyzer and with a Qubit 2.0 fluorometer using the Qubit dsDBNA HS Assay (Life Technologies), according to the manufacturer’s indications. The samples were then stored at −20 °C until subsequent sequencing.

#### 4.2.4. PGM Sequencing

The libraries previously prepared were pooled at 27 pM. Template preparation, ion sphere enrichment, and chip loading of an Ion 316 Chip Kit V2 were automatically set by an Ion Torrent Ion Chef system (ThermoFisher Scientific). High-throughput sequencing was based on the ion semi-conduction principle and operated by an Ion Personal Genome Machine (Ion PGM, ThermoFisher Scientific), using Ion PGM Hi-Q View Chef Reagents kits and Ion PGM Hi-Q Chef solutions kits. Raw data were analyzed with BD-NGS (Bioinformatics department, Institut Universitaire du Cancer IUCT, Oncopole, Toulouse, France), a software for the treatment and visualization of NGS data developed in our institute. The fastq primary files corresponding to each sample were generated by the sequencer. Then, BAM files were generated by aligning the reads to the hg19 human genome reference (GRCh37), and variant calling and annotations were performed by two variant callers in the pipeline: VarScan2 (2.3.7, Genome Institute, Washington University, Saint Louis, MO, USA) and HaplotypeCaller of the Genome Analysis Toolkit GATK (v.3.3.0, Broad Institute, Cambridge, MA, USA). Finally, the annotation step for VCF files was conducted with snpEff (v.4.3), snpSift (v.4.3), and the Ensembl variant predictor (VEP v.95.1) used as prediction tools for variant functional effect. The annotation databases used were Cosmic (v.87), Clinvar (v.20181028), GnomAD exomes (v.2.1), and dbSNP (v.151). After sorting the sequencing data according to the cohort minor allele frequency (MAF) ≥ 0.01, we visually inspected the remaining SNPs of interest using the Alamut software (v.2.11, Sofia Genetics, Boston, MA, USA).

### 4.3. Assessment of Liver Toxicity

In the TSAR trial, endpoints of toxicity were assessed and graded according to NCI-CTCAE v.4.0, including liver toxicity characterized by the measurement of biochemical liver parameters. Blood concentrations of AST/ALT, total plasma bilirubin, AP, and γ-GT, were recorded the first and fourth days of the treatment for cycles 1 and 2, and day 15 and day 21 for every cycle. Only variations of biological markers during cycles 1 and 2 were considered reflective of acute hepatotoxicity, for which severity was assessed as follows: elevations of AST/ALT, AP, and γ-GT over 5 times the upper limit normal (5x ULN) corresponded to a grade 3 adverse event and over 20x ULN reflected a grade 4 adverse event. Total blood bilirubin increased over 3x ULN was considered as a grade 3 elevation, and over 10x ULN as a grade 4 elevation. Severe cytolysis was considered for patients experiencing an AST/ALT increase ≥ grade 3. Cholestasis was assessed by grouping γ-GT, AP, and total plasma bilirubin elevations. Patients with isolated elevation of γ-GT were analyzed separately. Finally, overall hepatotoxicity was considered when patients presented at least one variation ≥ grade 3 of the hepatic biochemical parameters.

### 4.4. Statistical Analyses

Genetic analyses were performed with the package SNPassoc (v.1.9.2) of the R software (v.3.5.3) [59]. For each SNP, exact statistic tests were conducted to evaluate the deviation of the genotype frequencies from those expected under Hardy–Weinberg equilibrium (HWE), according to the method described by Wigginton et al. [60]. Only SNPs with HWE ≥ 0.05 and a genotyping rate higher than 80% were retained for the analysis. Population MAFs of the referenced SNPs were statistically compared with those of the Caucasian CEU (CentralEUrope, Utah residents CEPH (Centre d’Etude du Polymorphisme Humain) with Northern and Western European ancestry) population.

Preliminary to genotype/toxicity association statistical analyses, we performed chi-squared tests in order to evaluate the putative impact of prior treatment in hepatotoxicity. If significant, prior treatment would be included as an influencing variable in our subsequent genotype/toxicity association tests.

The univariate association analyses were performed between genotypes and the severe hepatic events previously detailed. For each genetic association, three logistic regression models were tested: (i) a codominant model assuming a per-allele effect that places heterozygous genotype midway between the two others homozygous genotypes (i.e., wild-type and variant), (ii) a dominant model grouping all carriers of the variant allele vs. wild-type carriers, and (iii) a recessive model comparing carriers of at least one wild-type allele and homozygous carriers of the variant [61]. Odds ratios (ORs) were calculated and the model with a significant raw *p*-value (i.e., *p <* 0.05) was retained as the most reliable result. Multiple test comparisons were conducted with the method of false discovery rate (FDR) according to the Benjamini–Hochberg (BH) procedure [62]. Finally, a haplotype study was performed, using Haploview software (v.4.2, Broad Institute, Cambridge, MA, USA) to visualize the patterns of linkage disequilibrium (LD) between SNPs and to select the haplotype blocks [63]. This step was followed by an analysis of regression models for binomial trait with Haplostats package (v.1.7.7) implemented in R. Regression coefficients and *p*-values for individual haplotype effects were estimated with the haplotype-specific generalized linear model (haplo.glm) function of Haplostats.

The findings were validated with a k-fold cross-validation procedure. A logistic regression was performed on two-thirds of the cohort (training dataset), and the obtained model was applied to the remaining third (validation dataset). Predictions were compared to observations by computation of the ROC’s (receiver operating characteristic curve) AUC. This procedure was repeated 10 times to obtain mean OR and AUC, as well as 95% confidence intervals assuming a Student’s *t* distribution with nine degrees of freedom.

## 5. Conclusions

This pharmacogenetic study is the first to describe the potential variants of interest involved in the trabectedin-related risk of severe hepatotoxicity in patients suffering from ASTS. In accordance with previous studies, this work provides assertions on the importance of functional ABC transporters and CYP450 in the disposition of trabectedin and/or its metabolites. Identification of deleterious variants of *ABCB1*, *ABCC2*, *ABCG2*, and *CYP3A5* brings new arguments in favor of the production of toxic metabolites that could be accumulated in the hepatocytes and have a toxic effect in situ.

The benefits of a pharmacogenetic investigation, which is a non-invasive and easy-to-perform approach, are consequent in terms of prevention of the risk of toxicity inherent in the use of trabectedin. Above all, even though ASTSs are rare diseases, predictive pharmacogenetic biomarkers could play an important role in the improvement of the prevention of the trabectedin-induced adverse drug reactions. This emphasizes the importance of validating these findings on larger cohorts of patients treated with trabectedin. If the role of these variants is confirmed in independent datasets, a prospective study—in which patients carrying these risk variants would have a more individualized treatment (reduced starting dose, enhanced surveillance of hepatic function)—could be conducted to evaluate the benefit in terms of reducing hepatic severe adverse events.

## Figures and Tables

**Table 1:** Patient characteristics and toxicity data.

Demographical Characteristics at Baseline		n (%) or (Range)
Total patients included in the pharmacogenetic study		63
Gender	Male	31 (49.2%)
	Female	32 (50.8%)
Age	Median (years)	65 (21.5–82.3)
Initial arm before crossover	Trabectedin	31 (49%)
	Best supportive care	32 (51%)
Baseline ECOG performance status	0	30 (47.6%)
	1	32 (50.8%)
	2	1 (1.6%)
**Tumor characteristics at baseline**		
Histological subtype of soft tissue sarcoma	STS-L: liposarcoma or leiomyosarcoma	35 (55.6%)
	STS-non L: other subtype	28 (44.4%)
Hepatic metastasis at baseline	Yes	13 (20.6%)
	No	50 (79.4%)
**Prior anticancer treatment before inclusion**		
Chemotherapy	Yes	63 (100%)
	No	0 (0%)
Neoadjuvant and/or adjuvant chemotherapy	Yes	21 (33.3%)
	No	42 (66.7%)
Lines of prior chemotherapy in advanced setting	Median	1 (0; 3)
	0	7 (11.1%)
	1	29 (46%)
	2	22 (34.9%)
	3	5 (7.9%)
Common prior chemotherapies in advanced setting	Anthracyclines	62 (98.4%)
	Ifosfamide	34 (54%)
	Gemcitabine	17 (27%)
	Dacarbazine	15 (23.8%)
	Pazopanib	10 (15.9%)
	Cyclophosphamide	5 (7.9%)
	Other	8 (12.7%)
**Trabectedin treatment after inclusion**		
Number of cycles administered per patient	Median	7 (1–33)
Total dose administered	Mean (mg)	2.25 (1.35–3.24)
**Treatment modification because of hepatotoxicity**		
Administration delay	Yes	6 (10%)
	No	57 (90%)
Dose reduction	Yes	17 (27%)
	No	46 (73%)
Stop of treatment	Yes	2 (3%)
	No	61 (97%)
**Hepatic side effects recorded during the two first courses of trabectedin**		
Cytolysis	Grades 0, 1, 2	36 (57%)
	Grades 3, 4	27 (43%)
Cholestasis	Grades 0, 1, 2	60 (95%)
	Grades 3, 4	3 (5%)
Isolated elevation of γ-GT	Grades 0, 1, 2	56 (89%)
	Grades 3, 4	7 (11%)
Overall hepatotoxicity	Grades 0, 1, 2	27 (43%)
	Grades 3, 4	36 (57%)

ECOG: Eastern Cooperative Oncology Group performance status; STS: soft tissue sarcoma; γ-GT: gamma-glutamyl transferases.

**Table 2:** Significant results (*p* < 0.05) of the univariate analysis conducted on 35 candidate single-nucleotide polymorphisms (SNPs) selected in the literature.

Gene	SNP	Genotype (*n*)	Severe HAE	% Severe HAE per Genotype	Genotype Comparison ^1^	*p*-Value	OR	95% CI	FDR
*ABCB1*	rs1128503 *c.1236C > T*	CC (19) CT (26) TT (14)	Cytolysis	68.4/34.6/35.7	CT-TT vs. **CC**	0.015	0.25	0.08–0.80	0.147
*ABCB1*	rs2032582 *c.2677G > T,A*	GG (18) GT/A (34) TT (11)	Overall hepatotoxicity	66.7/61.7/27.3	TT vs. **GG-GT**	0.027	0.22	0.05–0.91	0.705
*ABCC2*	rs17222723 *c.3563T > A*	TT (51) TA (9) AA (1)	Cytolysis	50.9/11.1/0	TA-AA vs. **TT**	0.010	0.11	0.01–0.91	0.147
*ABCC2*	rs2273697 *c.1249G > A*	GG (40) GA (21) AA (1)	Cytolysis	32.5/61.9/100	GA-AA vs. **GG**	0.018	3.63	1.22–10.83	0.147
*ABCC2*	rs2273697 *c.1249G > A*	GG (40) GA (21) AA (1)	Isolated elevation of γ-GT	17.5/0/0	GA-AA vs. **GG**	0.044	N/C	/	0.811
*ABCC2*	rs8187707 *c.4488C > T*	CC (50) CT (8) TT (0)	Cytolysis	48/12.5/0	CT vs. **CC**	0.045	0.15	0.02–1.35	0.371
*ABCC4*	rs9516519 c.*3261A > G	TT (42) TG (16) GG (1)	Overall hepatotoxicity	69/43.8/0	TG-GG vs. **TT**	0.048	0.31	0.10–1.01	0.444
*ABCG2*	rs7699188 *c.-15994C > T*	CC (31) CT (19) TT (2)	Cytolysis	32.3/63.2/50	CT-TT vs. **CC**	0.034	3.41	1.07–10.87	0.212
*CYP3A5*	rs776746 *c.6986A > G*	AA (2) AG (14) GG (47)	Overall hepatotoxicity	0/85.7/51.1	AA vs. GA vs. **GG**	0.012	5.75	1.16–28.55	0.403

^1^ Genotype in bold is considered as the reference for odds ratio comparison. FDR: false discovery rate; HAE: hepatic adverse effects; N/C: not calculable if, compared to other genotypes, 100% of the patients carrying the variant allele experienced HAE in the cohort; OR: odds ratio; SNP: single-nucleotide polymorphism; 95% CI: 95% confidence interval.

**Table 3:** Most significant results (*p <* 0.01) of the univariate analysis conducted on the whole database of 208 SNPs genotyped by NGS (Next-Generation Sequencing).

Gene	SNP	Genotype (n)	Severe HAE	% Severe HAE per Genotype	Genotype Comparison ^1^	*p*-Value	OR	95% CI	FDR
*ABCC2*	rs17216282 *c.4146 + 11G > C*	GG (50) GC (10) CC (0)	Cytolysis	52/10/0	GC vs. **GG**	0.009	0.1	0.01–0.87	0.698
*ABCC3*	rs2072365 *c.2714 + 29C > T*	CC (28) CT (29) TT (6)	Overall hepatotoxicity	39.2/79.3/33.3	CT vs. **CC** vs. TT	0.003	5.92	1.83–19.20	0.242
*ABCC3*	rs4148415 *c.2600-123C > T*	CC (27) CT (27) TT (5)	Overall hepatotoxicity	40.7/85.2/40	CT vs. **CC** vs. TT	0.001	8.36	2.26–31	0.242
*ABCC4*	rs11568647 *c.2213 + 108delC*	CC (53) Cdel (6) DelDel (0)	Overall hepatotoxicity	66/0/0	Cdel vs. **CC**	0.003	N/C	-	0.242
*ABCC4*	rs1751005 *c.1727 + 91G > A*	GG (43) GA (14) AA (2)	Cytolysis	55.8/14.3/50	GA-AA vs. **GG**	0.009	0.18	0.05–0.74	0.385
			Overall hepatotoxicity	72/28.6/50	GA-AA vs. **GG**	0.004	0.18	0.05–0.61	0.240
*ABCC4*	rs4148553 c.*694C > T	CC (23) CT (31) TT (6)	Cytolysis	34.8/41.9/100	TT vs. **CC-CT**	0.006	N/C	-	0.644

^1^ Genotype in bold is considered as the reference for odds ratio comparison. FDR: false discovery rate; HAE: hepatic adverse effects; N/C: not calculable if, compared to other genotypes, 100% of the patients carrying the variant allele experienced HAE in the cohort; OR: odds ratio; SNP: single-nucleotide polymorphism; 95% CI: 95% confidence interval.

## Supplementary Materials

The following are available online at https://www.mdpi.com/2072-6694/12/12/3647/s1: Table S1. Results of the k-fold cross-validation performed on significant SNPs presented in Table 2 and Table 3. Table S2. Significant results (*p <* 0.05) of the univariate analysis conducted on 208 SNPs genotyped by NGS. Figure S3. Association between haplotypes of seven SNPs in *ABCC2* gene and severe cytolysis. Table S4: Literature-based selection of candidate SNPs for primary analysis.
