# Supplementary material for: Pharmacogenetic Study of Trabectedin-Induced Severe Hepatotoxicity in Patients with Advanced Soft Tissue Sarcoma
Source: Cancers (Basel). 2020 Dec 4;12(12):3647. doi: 10.3390/cancers12123647 (PMC7761985; doi:10.3390/cancers12123647)
Supplement: Supplementary file 1 [file cancers-12-03647-s001.pdf]

cancers

MDPI

Supplementary Materials

Pharmacogenetic Study of Trabectedin-Induced Severe Hepatotoxicity in Patients with Advanced Soft Tissue Sarcoma

Maud Maillard [1,2,3], Christine Chevreau [3], Félicien Le Louedec [1,2,3], Manon Cassou [3], Caroline Delmas [1,3], Laure Gourdain [1,3], Jean-Yves Blay [4], Didier Cupissol [5], Emmanuelle Bompas [6], Antoine Italiano [7], Nicolas Isambert [8], Corinne Delcambre-Lair [9], Nicolas Penel [10], François Bertucci [11], Cécile Guillemet [12], Julien Plenecassagnes [3], Stéphanie Foulon [13,14], Étienne Chatelut [1,2,3], Axel Le Cesne [15] and Fabienne Thomas[1,2,3,]*

Table S1. Results of the k-fold cross-validation performed on significant SNPs presented in Table 2 and Table 3. Predictions were compared to observations by computation of the ROC curve's AUC. This procedure was repeated 10 times (K = 10) to obtain mean OR and AUC, as well as 95% confidence intervals assuming a Student's t-distribution with 9 degrees of freedom.

Gene	SNP	Hepatotoxicity endpoint	P value of the univariate analysis	OR of the univariate analysis	IC95 OR after K-cross	IC95 AUC after K-cross	K
ABCB1	rs1128503	Cytolysis	0.015	0.249	0.186 - 0.386	0.565 - 0.716	10
ABCB1	rs2032582	Overall hepatotoxicity	0.027	0.216	0.122 - 0.39	0.551 - 0.674	10
ABCC2	rs1722723	Cytolysis	0.01	0.107	0.073 - 0.228	0.578 - 0.677	10
ABCC2	rs2273697	Cytolysis	0.018	3.635	2.849 - 5.511	0.569 - 0.709	10
ABCC2	rs8187707	Cytolysis	0.045	0.155	0.118 - 0.359	0.569 - 0.651	10
ABCC4	rs9516519	Overall hepatotoxicity	0.048	0.314	0.22 - 0.455	0.541 - 0.688	10
ABCG2	rs7699188	Cytolysis	0.034	3.412	2.246 - 4.529	0.622 - 0.749	10
CYP3A5	rs776746	Overall hepatotoxicity	0.012	5.75	5.016 - 8.954	0.569 - 0.67	10
ABCC2	rs17216282	Cytolysis	0.009	0.1	0.073 - 0.191	0.603 - 0.668	10
ABCC3	rs2072365	Overall hepatotoxicity	0.003	5.92	4.984 - 6.574	0.674 - 0.748	10
ABCC3	rs4148415	Overall hepatotoxicity	0.001	8.36	6.574 - 8.752	0.737 - 0.802	10

ABC<br>C4	rs1156<br>8647	Overall<br>hepatotoxicity	0.003	*N/C*	*N/C*	*N/C*	1<br>0
ABC<br>C4	rs1751<br>005	Cytolysis	0.009	0.18	0.096 - 0.277	0.56 - 0.71	1<br>0
ABC<br>C4	rs1751<br>005	Overall<br>hepatotoxicity	0.004	0.18	0.117 - 0.263	0.593 - 0.749	1<br>0
ABC<br>C4	rs4148<br>553	Cytolysis	0.006	*N/C*	*N/C*	*N/C*	1<br>0

AUC: area under the receiver operating curve (ROC); IC95: 95% confidence interval; N/C: non calculable as no OR could be determined in the previous analyses (see Table 2 and Table 3); OR: odds-ratio; SNP: single-nucleotide polymorphism.

Table S2. Significant results (p <0.05) of the univariate analysis conducted on 208 SNPs genotyped by NGS.

Gene	SNP	Variant	Transcript and protein variation	Genotype (n)	Severe HAE	% Severe HAE per genotype	Genotype comparison[1]	P value	OR	95%CI	FDR
ABCB1	rs10276036	c.1000-44T>C	Intron variant	TT (20) / CT (26) / CC (13)	Cytolysis	65/34.6/38.5	CC-CT vs. **TT**	0.033	0.3	0.10-0.93	0.410
ABCB1	rs1128503	c.1236C>T	p.Gly412=	CC (19) / CT (26) / TT (14)	Cytolysis	68.4/34.6/35.7	CT-TT vs. **CC**	0.015	0.25	0.08-0.80	0.385
ABCB1	rs2032582	c.2677G>T	p.Ala893Thr	GG (18) / GT (34) / TT (11)	Overall hepatotoxicity	66.7/61.7/27.3	TT *vs.* **GG-GT**	0.027	0.22	0.05-0.91	0.902
ABCB1	rs2235013	c.1725+38C>T	Intron variant	CC (16) / CT (29) / TT (14)	Cytolysis	37.5/37.9/71.4	TT vs. **CC-CT**	0.026	4.12	1.11-15.21	1
ABCB1	rs2235033	c.1554+24A>G	Intron variant	AA (16) / AG (27) / GG (15)	Cytolysis	37.5/37/66.7	GG vs. **AG-GG**	0.048	3.37	0.98-11.65	1
ABCB1	rs2235046	c.2064+73T>C	Intron variant	CC (18) / CT (26) / TT (13)	Cytolysis	66.7/34.6/38.5	TC-TT vs. **CC**	0.029	0.28	0.09-091	0.410
ABCB1	rs4728699	c.2320-88T>C	Intron variant	TT (57) / CT (4) / CC (0)	Overall hepatotoxicity	63.2/0/0	CT vs. **TT**	0.024	0	-	0.840
ABCC2	rs1137968	c.4290G>T	p.Val1430=	GG (51) / GT (10) / TT (0)	Cytolysis	50.9/10/0	GT vs. **GG**	0.010	0.11	0.01-0.91	0.698
ABCC2	rs17216177	c.3742-34T>C	Intron variant	TT (50) / TC (8) / CC (0)	Cytolysis	52/12.5/0	TC vs. **TT**	0.027	0.13	0.02-1.15	0.698
ABCC2	rs17216212	c.4508+12G>A	Intron variant	GG (48) / GA (8) / AA (0)	Cytolysis	50/12.5/0	GA vs. **GG**	0.036	0.14	0.02-1.25	0.698
ABCC2	rs17216212	c.4508+12G>A	Intron variant	GG (48) / GA (8) / AA (0)	Overall hepatotoxicity	62.5/25/0	GA vs. **GG**	0.046	0.2	0.04-1.1	0.903
ABCC2	rs17216282	c.4146+11G>C	Intron variant	GG (50) / GC (10) / CC (0)	Cytolysis	52/10/0	GC vs. **GG**	0.009	0.1	0.01-0.87	0.698
ABCC2	rs17222723	c.3563T>A	p.Val1188Glu	TT (51) / TA (9) / AA (1)	Cytolysis	50.9/11.1/0	TA-AA vs. **TT**	0.010	0.11	0.01-0.91	0.698
ABCC2	rs2273697	c.1249G>A	p.Val417Ile	GG (40) / GA (21) / AA (1)	Cytolysis	32.5/61.9/100	GA-AA vs. **GG**	0.018	3.63	1.22-10.83	0.385
ABCC2	rs2273697	c.1249G>A	p.Val417Ile	GG (40) / GA (21) / AA (1)	Isolated elevation of gamma-GT	17.5/0/0	GA-AA vs. **GG**	0.044	0	-	0.604
ABCC2	rs41318031	c.2883+11C>T	Intron variant	CC (51) / CT (9) / TT (0)	Cytolysis	50.1/11.1/0	CT vs. **CC**	0.018	0.12	0.01-1.03	0.698
ABCC2	rs4148395	c.2621-139G>A	Intron variant	GG (38) / GA (20) / AA (1)	Cytolysis	34.2/65/100	GA-AA vs. **GG**	0.016	3.85	1.24-11.88	0.385
ABCC2	rs4148395	c.2621-139G>A	Intron variant	GG (38) / GA (20) / AA (1)	Isolated elevation of gamma-GT	18.4/0/0	GA-AA vs. **GG**	0.043	0	-	0.604
ABCC2	rs8187707	c.4488C>T	p.His1496=	CC (50) / CT (8) / TT (0)	Cytolysis	48/12.5/0	CT vs. **CC**	0.045	0.15	0.02-1.35	0.698
ABCC3	rs2072365	c.2714+29C>T	Intron variant	CC (28) / CT (29) / TT (6)	Cytolysis	32/58.6/16.7	CT vs. **CC** vs. TT	0.046	2.99	1.01-8.84	0.698
ABCC3	rs2072365	c.2714+29C>T	Intron variant	CC (28) / CT (29) / TT (6)	Overall hepatotoxicity	39.2/79.3/33.3	CT vs. **CC** vs. TT	0.003	5.92	1.83-19.20	0.242

ABCC3	rs4148415	c.2600-123C>T	Intron variant	CC (27) / CT (27) / TT (5)	Cytolysis	33.3/63/20	CT vs. **CC** vs. TT	0.041	3.4	1.11-10.40	0.698
ABCC3	rs4148415	c.2600-123C>T	Intron variant	CC (27) / CT (27) / TT (5)	Overall hepatotoxicity	40.7/85.2/40	CT vs. **CC** vs. TT	0.001	8.36	2.26-31	0.242
ABCC3	rs72837544	c.612+73C>A	Intron variant	CC (58) / CA (3) / AA (0)	Isolated elevation of gamma-GT	8.6/66.7/0	CA vs. **CC**	0.018	21.2	1.62-276.9	1
ABCC4	rs11568647	c.2213+108delC	Intron variant	CC (53) / CDel (6) / DelDel (0)	Cytolysis	51/0/0	Cdel vs. **CC**	0.027	0	-	0.698
ABCC4	rs11568647	c.2213+108delC	Intron variant	CC (53) / CDel (6) / DelDel (0)	Overall hepatotoxicity	66/0/0	Cdel vs. **CC**	0.003	0	-	0.242
ABCC4	rs1189437	c.2536-60T>G	Intron variant	TT (51) / TG (7) / GG (0)	Overall hepatotoxicity	54.9/100/0	TG vs. **TT**	0.035	0	-	0.903
ABCC4	rs1189466	c.2844G>A	p.Phe948=	GG (52) / GA (7) / AA (0)	Overall hepatotoxicity	55.8/100/0	AG vs. **GG**	0.036	0	-	0.903
ABCC4	rs1751005	c.1727+91G>A	Intron variant	GG (43) / GA (14) / AA (2)	Cytolysis	55.8/14.3/50	GA-AA vs. **GG**	0.009	0.18	0.05-0.74	0.385
ABCC4	rs1751005	c.1727+91G>A	Intron variant	GG (43) / GA (14) / AA (2)	Overall hepatotoxicity	72/28.6/50	GA-AA vs. **GG**	0.004	0.18	0.05-0.61	0.240
ABCC4	rs2274408	c.911+80T>C	Intron variant	TT (15) / TC (29) / CC (8)	Isolated elevation of gamma-GT	6.7/6.9/37.5	CC vs. **TT-TC**	0.030	8.2	1.29-52.16	1
ABCC4	rs2274408	c.911+80T>C	Intron variant	TT (15) / TC (29) / CC (8)	Overall hepatotoxicity	33.3/65.5/75	TC-CC vs. **TT**	0.024	4.17	1.16-14.91	0.424
ABCC4	rs2296653	c.1727+118A>G	Intron variant	AA (16) / GA (27) / GG (16)	Cytolysis	62.5/48.1/25	GG vs. **AA-AG**	0.046	0.29	0.08-1.04	1
ABCC4	rs2296653	c.1727+118A>G	Intron variant	AA (16) / GA (27) / GG (16)	Overall hepatotoxicity	81.3/59.3/43.8	GA-GG vs. **AA**	0.044	0.27	0.07-1.07	0.424
ABCC4	rs4148553	c.*694C>T	3'UTR variant	CC (23) / CT (31) / TT (6)	Cytolysis	34.8/41.9/100	TT vs. **CC-CT**	0.006	N/C	-	0.644
ABCC4	rs6650282	c.*168A>G	3'UTR variant	AA (11) / AG (32) / GG (14)	Cytolysis	21.4/50/63.6	AG-AA vs. **GG**	0.031	4.22	1.03-17.28	0.410
ABCC4	rs6650282	c.*168A>G	3'UTR variant	AA (11) / AG (32) / GG (14)	Overall hepatotoxicity	35.7/65.6/72.7	AG-AA vs. **GG**	0.037	3.73	1.05-13.22	0.424
ABCC4	rs9516519	c.*3261T>G	3'UTR variant	TT (42) / TG (16) / GG (1)	Overall hepatotoxicity	69/43.8/0	TG-GG vs. **TT**	0.048	0.31	0.10-1.01	0.424
ABCG2	rs7699188	c.-15994C>T	Intron variant	CC (31) / CT (19) / TT (2)	Cytolysis	32.3/63.2/50	CT-TT vs. **CC**	0.034	3.41	1.07-10.87	0.410
CYP2D6	rs29001678	c.180+90G>A	Intron variant	GG (55) / GA (5) / AA (1)	Cytolysis	40/80/100	GA-AA vs. **GG**	0.038	7.5	0.83-68.63	0.410
CYP2E1	rs2249694	c.1298-131G>A	Intron variant	GG (30) / GA (23) / AA (3)	Isolated elevation of gamma-GT	20/0/0	AG-AA vs. **GG**	0.025	0	-	0.553
CYP2E1	rs2249694	c.1298-131G>A	Intron variant	GG (30) / GA (23) / AA (3)	Overall hepatotoxicity	70/43.5/33.3	AG-AA vs. **GG**	0.036	0.31	0.10-0.95	0.424
CYP2E1	rs2249695	c.1298-116C>T	Intron variant	CC (30) / CT (24) / TT (3)	Isolated elevation of gamma-GT	20/0/0	TC-TT vs. **CC**	0.025	0	-	0.553
CYP2E1	rs2249695	c.1298-116C>T	Intron variant	CC (30) / CT (24) / TT (3)	Overall hepatotoxicity	70/45.8/33.3	TC-TT vs. **CC**	0.050	0.34	0.12-1.02	0.424

CYP2E1	rs2480256	c.*46G>A	3'UTR variant	GG (35) / GA (20) / AA (4)	Isolated elevation of gamma-GT	20/0/0	AG-AA vs. **GG**	0.035	0	-	0.604
CYP2E1	rs2480256	c.*46G>A	3'UTR variant	GG (35) / GA (20) / AA (4)	Overall hepatotoxicity	71.4/50/25	AG-AA vs. **GG**	0.048	0.34	0.11-1	0.424
CYP2E1	rs2480257	c.*41T>A	3'UTR variant	TT (33) / TA (0) / AA (0)	Isolated elevation of gamma-GT	21.2/0/0	TA-AA vs. **TT**	0.014	0	-	0.553
CYP2E1	rs2480258	c.1298-184C>T	Intron variant	CC (30) / TC (23) / TT (3)	Isolated elevation of gamma-GT	20/0/0	TC-TT vs. **CC**	0.025	0	-	0.553
CYP2E1	rs2480258	c.1298-184C>T	Intron variant	CC (30) / TC (23) / TT (3)	Overall hepatotoxicity	70/43.5/33.3	TC-TT vs. **CC**	0.036	0.31	0.10-0.95	0.424
CYP2E1	rs2480259	c.1298-208G>A	Intron variant	GG (30) / GA (23) / AA (3)	Isolated elevation of gamma-GT	20/0/0	AG-AA vs. **GG**	0.025	0	-	0.553
CYP2E1	rs2480259	c.1298-208G>A	Intron variant	GG (30) / GA (23) / AA (3)	Overall hepatotoxicity	70/43.5/33.3	AG-AA vs. **GG**	0.036	0.31	0.10-0.95	0.424
CYP3A5	rs776746	c.6986A>G	Splicing acceptor variant	AA (2) / AG (14) / GG (47)	Overall hepatotoxicity	0/85.7/51.1	TC vs. **CC** vs. TT	0.012	5.75	1.16-28.55	0.605

[1] Genotype in bold is considered as the reference for odds ratio comparison. FDR: false-discovery rate; HAE: hepatic adverse effects; N/C: not calculable if compared to other genotypes, 100% of the patients carrying the variant allele experienced HAE in the cohort; OR: odds ratio; SNP: single-nucleotide polymorphism; 95%CI: 95% confidence interval.

Figure S3. Association between haplotypes of seven SNPs in *ABCC2* gene and severe cytolysis. Three models are described (additive, dominant, recessive). The dot line represents the significant p-value threshold of global score test expressed in negative logarithm (log (p) = 1.3).

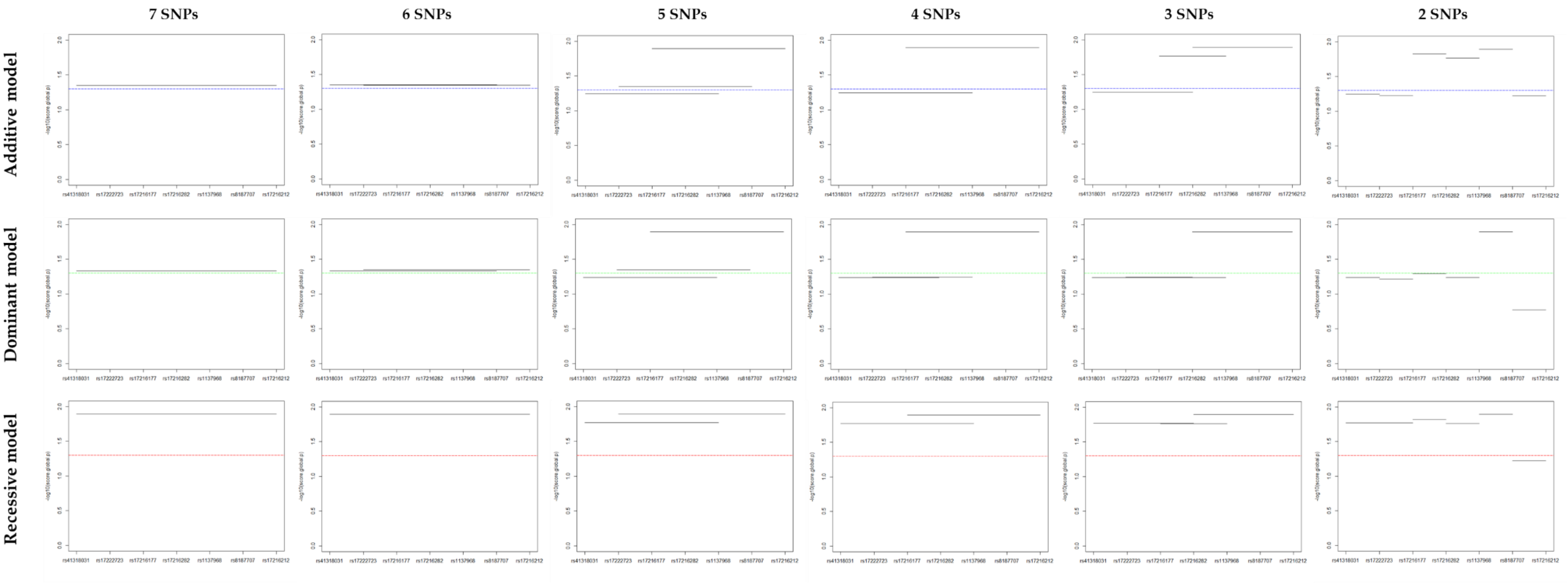

Table S4. Literature-based selection of candidate SNPs for primary analysis. HWE: Hardy-Weinberg equilibrium; N/I: not indicated; SNP: single nucleotide polymorphism; Ref.: references; ↓: decreased; ↑: increased.

Gene	Variant	Genetic sequence variation	Transcript and protein variation	Functional impact	HWE P > 0.05	Genotyping rate >80%	Candidate SNP for primary analysis	Ref.
ABCB1	rs1045642	c.3435C>T	p.Ile1145=	↓ *ABCB1* expression. ↓ P-gp activity. ↓ liver toxicity in patients carrying the wild-type allele.	Yes	Yes	*	[1,2]
ABCB1	rs1128503	c.1236C>T	p.Gly412=	Conflicting results. ↑ or ↓ extracellular efflux and may influence exposure to drug and toxicity.	Yes	Yes	*	[2–5]
ABCB1	rs2032582	c.2677G>T,A	p.Ser893Ala/Thr	Controversial variant. ↑ or ↓ activity of P-gp and efflux of its substrates.	Yes	Yes	*	[6–8]
ABCB1	rs2032583	g.87531245T>C	Intron variant	Associated with a higher frequency of adverse-effects probably due to a specific-serotonergic reuptake inhibitors accumulation.	Yes	Yes	*	[9]
ABCC2	rs17222723	c.3563T>A	p.Val1188Glu	Allele A ↑ MRP2 expression in liver of patients undergoing liver resection. Functional study in transfected Flp-In HEK293 cells	Yes	Yes	*	[10,11]

ABCC2	rs2273697	c.1249G>A	p.Val417Ile	showed increased activity of MRP2. Conflicting results. Normal or ↑ expression of MRP2 in A-allele carriers *versus* wild-type individuals. ↓ efflux transport of MRP2 substrates and carbamazepine.	Yes	Yes	*	[10,12–14]

Table S4 (continued). Literature-based selection of candidate SNPs for primary analysis. HWE: Hardy-Weinberg equilibrium; N/I: not indicated; SNP: single nucleotide polymorphism; Ref.: references; ↓: decreased; ↑: increased.

Gene	Variant	Genetic sequence variation	Transcript and protein variation	Functional impact	HWE P > 0.05	Genotyping rate >80%	Candidate SNP for primary analysis	Ref.
ABCC2	rs3740066	c.3972C>T	p.Ile1324Met	Probable effect in splicing control. ↑ the risk of vincristine-induced neurotoxicity. ↑ methotrexate plasma concentrations.	Yes	No		[15]
ABCC2	rs717620	c.-24C>T	5'UTR variant	Conflicting results. T allele may up regulate the expression of MRP2. ↑ methotrexate, diclofenac and trabectedin toxicities. ↓ exposure to irinotecan. In haplotype, associated with a higher	Yes	Yes	*	[14,16–20]

				risk of toxic hepatitis.				
ABCC2	rs8187707	c.4488C>T	p.His1496=	Identified in the lethal-case following trabectedin chemotherapy. Associated with a ↓ creatinine clearance during tenofovir treatment.	Yes	Yes	*	[16,21]
ABCC2	rs8187710	c.4544G>A	p.Cys1515Tyr	Identified in the lethal-case following trabectedin chemotherapy. ↑ gastrointestinal toxicity of cisplatin when combined with taxanes. Variant associated with a ↓ efflux of lopinavir in kidney cells and a decreased clearance of carboplatin.	Yes	Yes	*	[22,23]
ABCC3	rs1051640	c.4509A>G	p.Glu1503=	↑ the risk of cisplatin cumulative ototoxicity.	Yes	Yes	*	[24]

Table S4 (continued). Literature-based selection of candidate SNPs for primary analysis. HWE: Hardy-Weinberg equilibrium; N/I: not indicated; SNP: single nucleotide polymorphism; Ref.: references; ↓: decreased; ↑: increased.

Gene	Variant	Genetic sequence variation	Transcript and protein variation	Functional impact	HWE P > 0.05	Genotyping rate >80%	Candidate SNP for primary analysis	Ref.
ABCC3	rs4148416	c.3039C>T	p.Gly1013=	No functional study for this variant. Allele T ↑ the risk of death when treated with cisplatin, cyclophosphamide, doxorubicin, methotrexate and vincristine in people with osteosarcoma.	Yes	Yes	*	[25]
ABCC3	rs4793665	c.-211C>T	Promoter variant	↓ promoter activity. ↓ gene transcription in mRNA.	Yes	Yes	*	[26–29]
ABCC4	rs1059751	c.*879T>C	3'UTR variant	↑ the risk of antiretroviral drug toxicity.	Yes	Yes	*	[30]
ABCC4	rs11568658	c.559G>T	p.Gly187Trp	↑ the risk of antiretroviral drug toxicity.	Yes	Yes	*	[31]
ABCC4	rs1678387	g.240795A>G	Intron variant	↑ the risk of osteonecrosis in G patients treated with biphosphonates.	*N/I*	*N/I*		[32]
ABCC4	rs17268282	g.33387C>A	Intron variant	Modulation of furosemide response in patients with decompensated heart failure.	*N/I*	*N/I*		[33]
ABCC4	rs1751034	c.3348G>C/A	p.Lys1116Lys	↑ antiretroviral drugs plasma concentration.	Yes	No		[34]
ABCC4	rs2274407	c.912G>T	p.Lys304Asn	Impaired splicing of ABCC4 mRNA. Transport function not altered. T allele associated with worse disease-free survival in patients with acute	Yes	Yes	*	[35]

ABCC4	rs3742106	c.*38T>G	3'UTR variant	lymphoblastic leukemia. ↑ antiretroviral drugs plasma concentration.	Yes	Yes	*	[36,37]

Table S4. (continued). Literature-based selection of candidate SNPs for primary analysis. HWE: Hardy-Weinberg equilibrium; N/I: not indicated; SNP: single nucleotide polymorphism; Ref.: references; ↓: decreased; ↑: increased.

**Gene**	**Variant**	**Genetic sequence variation**	**Transcript and protein variation**	**Functional impact**	**HWE P > 0.05**	**Genotyping rate >80%**	**Candidate SNP for primary analysis**	**Ref.**
ABCC4	rs3765534	c.2269G>A	p.Glu682Lys	↑ risk of leukopenia in patients treated with azathioprine/6-mercaptopurine. ↑ 6-thioguanine intracellular concentrations.	Monomorphic	Yes		[38]
ABCC4	rs7317112	g.35178T>C	Intron variant	Wild-type allele ↑ the risk of mucositis in methotrexate-treated pediatrics.	*N/I*	*N/I*		[39]
ABCC4	rs9516519	c.*3261T>G	3'UTR variant	Allele C induces the loss of a micro-RNA binding site, resulting in an increased expression of *ABCC4* mRNA. Wild-type allele T ↑ plasma concentrations of methotrexate in pediatric ALL patients.	Yes	Yes	*	[40]
ABCC4	rs9561765	g.275458C>T	Intron variant	↑ response to imatinib in CT genotype patients treated for gastrointestinal stromal tumors.	*N/I*	*N/I*		[41]

ABCC4	rs9561778	g.244986C>T	Intron variant	↑ risk of cyclophosphamide-induced hematotoxicity and digestive toxicity.	*N/I*	*N/I*		[42]
ABCG2	rs2231137	c.34G>A	p.Val12Met	Aberrant splicing variant. ↓ *ABCG2* expression in liver. Impaired BCRP localization in hepatocyte membrane. ↓ dose of imatinib for A carriers. ↑ irinotecan-induced severe diarrhea.	Yes	Yes	*	[43–46]
ABCG2	rs2231142	c.421C>A	p.Glu141Lys	↓ expression of BCRP in apical cell membrane.	Yes	Yes	*	[47]
ABCG2	rs7699188	c.-15994C>T	Intron variant	↑ BCRP mRNA in liver. ↑ oral clearance of imatinib. ↑ risk of irinotecan-related non-hematological toxicity.	Yes	Yes	*	[43,48]
CYP2C9	rs1057910	c.1075A>G (*3)	p.Ile359Val	↓ CYP2C9 activity.	Yes	Yes	*	[49]
CYP2C9	rs1799853	c.430C>T (*2)	p.Cys144Arg	↓ CYP2C9 activity.	Yes	Yes	*	[50]
CYP2C19	rs12248560	c.-806C>T (*17)	Intron variant	Associated with ultra-fast metabolizer phenotype.	Yes	Yes	*	[51]
CYP2C19	rs12769205	c.332-23A>G (*2)	Intron variant	Nonfunctional CYP2C19 caused by splicing defect.	Yes	Yes	*	[52]
CYP2C19	rs3758581	c.991G>A	p.Val331Ile	↓ CYP2C19 function.	Yes	Yes	*	[53]
CYP2C19	rs4244285	c.681G>A (*2)	p.Pro227=	Aberrant splice site insertion; associated with poor metabolizer phenotype.	Yes	Yes	*	[54]

Table S4. (continued). Literature-based selection of candidate SNPs for primary analysis. HWE: Hardy-Weinberg equilibrium; N/I: not indicated; SNP: single nucleotide polymorphism; Ref.: references; ↓: decreased; ↑: increased.

Gene	Variant	Genetic sequence variation	Transcript and protein variation	Functional impact	HWE P > 0.05	Genotyping rate >80%	Candidate SNP for primary analysis	Ref.
CYP2D6	rs16947	c.2850C>T,A (*2)	p.Arg296Cys/Ser	Amplification of *CYP2D6* locus associated with ultra-fast metabolizer phenotype.	Yes	Yes	*	[55]
CYP2D6	rs1065852	c.100C>T (*10)	p.Pro34Ser	Nonfunctional variant. ↓ or abolishment of CYP2D6 activity. Having two copies defines the poor metabolizer phenotype.	No	Yes		[56]
CYP2D6	rs1135840	c.4180G>C	p.Ser486Thr	GG genotype ↑ the risk of antituberculosis drug-induced hepatotoxicity.	No	Yes		[57]
CYP2D6	rs28371725	c.2988G>A (*41)	p.Gly309Glu	Splicing defect. ↓ CYP2D6 activity.	Yes	Yes	*	[58]
CYP2D6	rs35742686	c.2549delA (*3)	Frameshift variant	Nonfunctional variant.	Yes	Yes	*	[59]
CYP2D6	rs5030655	c.454delA (*6)	Frameshift variant	Nonfunctional variant. ↓ or abolishment of CYP2D6 activity. Having two copies defines the poor metabolizer phenotype.	*N/I*	*N/I*		[60]
CYP2D6	rs5030656	c.841_843delAAG (*9)	p.Lys281del	↓ CYP2D6 activity.	Yes	Yes	*	[61]
CYP2E1	rs2515641	c.1263C>T	p.Phe421=	T allele ↓ the hepatotoxicity of various anticancer drugs.	Yes	Yes	*	[62]
CYP2E1	rs6413420	c.-71G>T (*7)	Non coding variant	↑ transcription and activation of *CYP2E1*.	Yes	Yes	*	[63]
CYP3A4	rs2242480	g.20239G>A (*1G)	Intron variant	↓ CYP3A4 activity.	Yes	Yes	*	[64]
CYP3A4	rs2740574	c.-392G>A (*1B)	Promoter variant	↑ CYP3A4 activity.	No	Yes		[65]

Table S4. (continued). Literature-based selection of candidate SNPs for primary analysis. HWE: Hardy-Weinberg equilibrium; N/I: not indicated; SNP: single nucleotide polymorphism; Ref.: references; ↓: decreased; ↑: increased.

CYP3A4	rs35599367	c.15389C>T	Intron variant	Altered RNA splicing. ↓ gene transcription and activity of the CYP3A4 in liver.	Yes	Yes	*	[66,67]
CYP3A5	rs776746	c.6986A>G	Splicing acceptor variant	Aberrant splice site insertion. Allele G (*3, most frequent) is associated with a nonfunctional enzyme. Carriers of *1 allele have an active enzyme and may be exposed to an increased metabolism and drug-related toxicity.	Yes	Yes	*	[68–70]

Additional references

1. Kimchi-Sarfaty, C.; Marple, A.H.; Shinar, S.; Kimchi, A.M.; Scavo, D.; Roma, M.I.; Kim, I.-W.; Jones, A.; Arora, M.; Gribar, J.; et al. Ethnicity-related polymorphisms and haplotypes in the human ABCB1 gene. *Pharmacogenomics* **2007**, *8*, 29–39, doi:10.2217/14622416.8.1.29.
2. Salama, N.N.; Yang, Z.; Bui, T. o. t.; Ho, R.J.Y. MDR1 haplotypes significantly minimize intracellular uptake and transcellular P-gp substrate transport in recombinant LLC-PK1 cells. *J. Pharm. Sci.* **2006**, *95*, 2293–2308, doi:10.1002/jps.20717.
3. Wolking, S.; Schaeffeler, E.; Lerche, H.; Schwab, M.; Nies, A.T. Impact of Genetic Polymorphisms of ABCB1 (MDR1, P-Glycoprotein) on Drug Disposition and Potential Clinical Implications: Update of the Literature. *Clin. Pharmacokinet.* **2015**, *54*, 709–735, doi:10.1007/s40262-015-0267-1.
4. Beuselinck, B.; Lambrechts, D.; Van Brussel, T.; Wolter, P.; Cardinaels, N.; Joniau, S.; Lerut, E.; Karadimou, A.; Couchy, G.; Sebe, P.; et al. Efflux pump ABCB1 single nucleotide polymorphisms and dose reductions in patients with metastatic renal cell carcinoma treated with sunitinib. *Acta Oncol.* **2014**, *53*, 1413–1422, doi:10.3109/0284186X.2014.918276.
5. Giacomelli, A.; Riva, A.; Falvella, F.S.; Oreni, M.L.; Cattaneo, D.; Cheli, S.; Renisi, G.; Cristo, V.D.; Lupo, A.; Clementi, E.; et al. Clinical and genetic factors associated with increased risk of severe liver toxicity in a monocentric cohort of HIV positive patients receiving nevirapine-based antiretroviral therapy. *BMC Infect. Dis.* **2018**, *18*, 1–8, doi:10.1186/s12879-018-3462-5.
6. Hodges, L.M.; Markova, S.M.; Chinn, L.W.; Gow, J.M.; Kroetz, D.L.; Klein, T.E.; Altman, R.B. Very important pharmacogene summary: ABCB1 (MDR1, P-glycoprotein). *Pharmacogenet. Genomics* **2011**, *21*, 152–161, doi:10.1097/FPC.0b013e32833385a1c.
7. Kim, R.B.; Leake, B.F.; Choo, E.F.; Dresser, G.K.; Kubba, S.V.; Schwarz, U.I.; Taylor, A.; Xie, H.-G.; McKinsey, J.; Zhou, S.; et al. Identification of functionally variant MDR1 alleles among European Americans and African Americans. *Clin. Pharmacol. Ther.* **2001**, *70*, 189–199, doi:10.1067/mcp.2001.117412.
8. Llaudó, I.; Colom, H.; Giménez-Bonafé, P.; Torras, J.; Caldés, A.; Sarrias, M.; Cruzado, J.M.; Oppenheimer, F.; Sánchez-Plumed, J.; Gentil, M.Á.; et al. Do drug transporter (ABCB1) SNPs and P-glycoprotein function influence cyclosporine and macrolides exposure in renal transplant patients? Results of the pharmacogenomic substudy within the symphony study. *Transpl. Int.* **2013**, *26*, 177–186, doi:10.1111/tri.12018.
9. de Klerk, O.L.; Nolte, I.M.; Bet, P.M.; Bosker, F.J.; Snieder, H.; den Boer, J.A.; Bruggeman, R.; Hoogendijk, W.J.; Penninx, B.W. ABCB1 gene variants influence tolerance to selective serotonin reuptake inhibitors in a large sample of Dutch cases with major depressive disorder. *Pharmacogenomics J.* **2013**, *13*, 349–353, doi:10.1038/tpj.2012.16.
10. Wen, X.; Joy, M.S.; Aleksunes, L.M. In Vitro Transport Activity and Trafficking of MRP2/ABCC2 Polymorphic Variants. *Pharm. Res.* **2017**, *34*, 1637–1647, doi:10.1007/s11095-017-2160-0.
11. Meier, Y.; Pauli-Magnus, C.; Zanger, U.M.; Klein, K.; Schaeffeler, E.; Nussler, A.K.; Nussler, N.; Eichelbaum, M.; Meier, P.J.; Stieger, B. Interindividual variability of canalicular ATP-binding-cassette (ABC)–transporter expression in human liver. *Hepatology* **2006**, *44*, 62–74, doi:10.1002/hep.21214.
12. Megaraj, V.; Zhao, T.; Paumi, C.M.; Gerk, P.M.; Kim, R.; Vore, M. Functional Analysis of Non-synonymous Single Nucleotide Polymorphisms of Multidrug Resistance Protein 2 (MRP2; ABCC2). *Pharmacogenet. Genomics* **2011**, *21*, 506–515, doi:10.1097/FPC.0b013e328348c786.
13. Kim, W.-J.; Lee, J.H.; Yi, J.; Cho, Y.-J.; Heo, K.; Lee, S.H.; Kim, S.W.; Kim, M.-K.; Kim, K.H.; In Lee, B.; et al. A nonsynonymous variation in MRP2/ABCC2 is associated with neurological adverse drug reactions of carbamazepine in patients with epilepsy: *Pharmacogenet. Genomics* **2010**, 1, doi:10.1097/FPC.0b013e328338073a.
14. Deo, A.K.; Prasad, B.; Balogh, L.; Lai, Y.; Unadkat, J.D. Interindividual Variability in Hepatic Expression of the Multidrug Resistance-Associated Protein 2 (MRP2/ABCC2): Quantification by Liquid Chromatography/Tandem Mass Spectrometry. *Drug Metab. Dispos.* **2012**, *40*, 852–855, doi:10.1124/dmd.111.043810.
15. Lopez-Lopez, E.; Gutierrez-Camino, A.; Astigarraga, I.; Navajas, A.; Echebarria-Barona, A.; Garcia-Miguel, P.; Garcia de Andoin, N.; Lobo, C.; Guerra-Merino, I.; Martin-Guerrero, I.; et al. Vincristine pharmacokinetics pathway and neurotoxicity during early phases of treatment in pediatric acute lymphoblastic leukemia. *Pharmacogenomics* **2016**, *17*, 731–741, doi:10.2217/pgs-2016-0001.

16. Laurenty, A.-P.; Thomas, F.; Chatelut, E.; Bétrian, S.; Guellec, C.L.; Hennebelle, I.; Guellec, S.L.; Chevreau, C. Irreversible hepatotoxicity after administration of trabectedin to a pleiomorphic sarcoma patient with a rare *ABCC2* polymorphism: a case report. *Pharmacogenomics* **2013**, *14*, 1389–1396, doi:10.2217/pgs.13.124.
17. Barraclough, K.A.; Lee, K.J.; Staatz, C.E. Pharmacogenetic influences on mycophenolate therapy. *Pharmacogenomics* **2010**, *11*, 369–390, doi:10.2217/pgs.10.9.
18. Franke, R.; Lancaster, C.; Peer, C.; Gibson, A.; Kosloske, A.; Orwick, S.; Mathijssen, R.; Figg, W.; Baker, S.; Sparreboom, A. Dependence of erythromycin metabolism on ABCC2 (MRP2) transport function. *Clin. Pharmacol. Ther.* **2011**, *89*, 693–701, doi:10.1038/clpt.2011.25.
19. Nguyen, T.D.; Markova, S.; Liu, W.; Gow, J.M.; Baldwin, R.M.; Habashian, M.; Relling, M.V.; Ratain, M.J.; Kroetz, D.L. Functional characterization of ABCC2 promoter polymorphisms and allele specific expression. *Pharmacogenomics J.* **2013**, *13*, 396–402, doi:10.1038/tpj.2012.20.
20. Choi, J.H.; Ahn, B.M.; Yi, J.; Lee, J.H.; Lee, J.H.; Nam, S.W.; Chon, C.Y.; Han, K.; Ahn, S.H.; Jang, I.; et al. Mrp2 haplotypes confer differential susceptibility to toxic liver injury. *Pharmacogenet. Genomics* **2007**, *17*, 403–415, doi:10.1097/01.fpc.0000236337.41799.b3.
21. Dahlin, A.; Wittwer, M.; de la Cruz, M.; Woo, J.M.; Bam, R.; Scharen-Guivel, V.; Flaherty, J.; Ray, A.S.; Cihlar, T.; Gupta, S.K.; et al. A Pharmacogenetic Candidate Gene Study of Tenofovir-Associated Fanconi Syndrome. *Pharmacogenet. Genomics* **2015**, *25*, 82–92, doi:10.1097/FPC.0000000000000110.
22. Elens, L.; Tyteca, D.; Panin, N.; Courtoy, P.; Lison, D.; Demoulin, J.-B.; Haufroid, V. Functional defect caused by the 4544G>A SNP in ABCC2: potential impact for drug cellular disposition. *Pharmacogenet. Genomics* **2011**, *21*, 884–893, doi:10.1097/FPC.0b013e32834d672b.
23. Gao, B.; Lu, Y.; Nieuweboer, A.J.M.; Xu, H.; Beesley, J.; Boere, I.; de Graan, A.-J.M.; de Bruijn, P.; Gurney, H.; J. Kennedy, C.; et al. Genome-wide association study of paclitaxel and carboplatin disposition in women with epithelial ovarian cancer. *Sci. Rep.* **2018**, *8*, 1508, doi:10.1038/s41598-018-19590-w.
24. Pussegoda, K.; Ross, C.J.; Visscher, H.; Yazdanpanah, M.; Brooks, B.; Rassekh, S.R.; Zada, Y.F.; Dubé, M.-P.; Carleton, B.C.; Hayden, M.R. Replication of TPMT and ABCC3 Genetic Variants Highly Associated With Cisplatin-Induced Hearing Loss in Children. *Clin. Pharmacol. Ther.* **2013**, *94*, 243–251, doi:10.1038/clpt.2013.80.
25. Caronia, D.; Patiño-Garcia, A.; Peréz-Martínez, A.; Pita, G.; Moreno, L.T.; Zalacain-Díez, M.; Molina, B.; Colmenero, I.; Sierrasesúmaga, L.; Benítez, J.; et al. Effect of ABCB1 and ABCC3 Polymorphisms on Osteosarcoma Survival after Chemotherapy: A Pharmacogenetic Study. *PLoS ONE* **2011**, *6*, e26091, doi:10.1371/journal.pone.0026091.
26. Lang, T.; Hitzl, M.; Burk, O.; Mornhinweg, E.; Keil, A.; Kerb, R.; Klein, K.; Zanger, U.; Eichelbaum, M.; Fromm, M. Genetic polymorphisms in the multidrug resistance-associated protein 3 (ABCC3, MRP3) gene and relationship to its mRNA and protein expression in human liver. *Pharmacogenetics* **2004**, *14*, 155–164, doi:10.1097/00008571-200403000-00003.
27. Hegyi, M.; Arany, A.; Semsei, A.F.; Csordas, K.; Eipel, O.; Gezsi, A.; Kutszegi, N.; Csoka, M.; Muller, J.; Erdelyi, D.J.; et al. Pharmacogenetic analysis of high-dose methotrexate treatment in children with osteosarcoma. *Oncotarget* **2016**, *8*, 9388–9398, doi:10.18632/oncotarget.11543.
28. Bruhn, O.; Cascorbi, I. Polymorphisms of the drug transporters ABCB1, ABCG2, ABCC2 and ABCC3 and their impact on drug bioavailability and clinical relevance. *Expert Opin. Drug Metab. Toxicol.* **2014**, *10*, 1337–1354, doi:10.1517/17425255.2014.952630.
29. Errasti-Murugarren, E.; Pastor-Anglada, M. Drug transporter pharmacogenetics in nucleoside-based therapies. *Pharmacogenomics* **2010**, *11*, 809–841, doi:10.2217/pgs.10.70.
30. Likanonsakul, S.; Suntisuklappon, B.; Nitiyanontakij, R.; Prasithsirikul, W.; Nakayama, E.E.; Shioda, T.; Sangsajja, C. A Single-Nucleotide Polymorphism in ABCC4 Is Associated with Tenofovir-Related Beta2-Microglobulinuria in Thai Patients with HIV-1 Infection. *PLOS ONE* **2016**, *11*, e0147724, doi:10.1371/journal.pone.0147724.
31. Billat, P.-A.; Ossman, T.; Saint-Marcoux, F.; Essig, M.; Rerolle, J.-P.; Kamar, N.; Rostaing, L.; Kaminski, H.; Fabre, G.; Otyepka, M.; et al. Multidrug resistance-associated protein 4 (MRP4) controls ganciclovir intracellular accumulation and contributes to ganciclovir-induced neutropenia in renal transplant patients. *Pharmacol. Res.* **2016**, *111*, 501–508, doi:10.1016/j.phrs.2016.07.012.
32. Nicoletti P.; Cartsos V.M.; Palaska P.K.; Shen Y.; Floratos A.; Zavras A.I. Genomewide Pharmacogenetics of Bisphosphonate-Induced Osteonecrosis of the Jaw: The Role of RBMS3. *The Oncologist* **2012**, *17*, 279–287, doi:10.1634/theoncologist.2011-0202.

33. de Denus, S.; Rouleau, J.L.; Mann, D.L.; Huggins, G.S.; Cappola, T.P.; Shah, S.H.; Keleti, J.; Zada, Y.F.; Provost, S.; Bardhadi, A.; et al. A pharmacogenetic investigation of intravenous furosemide in decompensated heart failure: a meta-analysis of three clinical trials. *Pharmacogenomics J.* **2017**, *17*, 192–200, doi:10.1038/tpj.2016.4.
34. Kiser, J.J.; Aquilante, C.L.; Anderson, P.L.; King, T.M.; Carten, M.L.; Fletcher, C.V. Clinical and genetic determinants of intracellular tenofovir diphosphate concentrations in HIV-infected patients. *J. Acquir. Immune Defic. Syndr. 1999* **2008**, *47*, 298–303, doi:10.1097/qai.0b013e31815e7478.
35. Mesrian Tanha, H.; Rahgozar, S.; Mojtabavi Naeini, M. ABCC4 functional SNP in the 3′ splice acceptor site of exon 8 (G912T) is associated with unfavorable clinical outcome in children with acute lymphoblastic leukemia. *Cancer Chemother. Pharmacol.* **2017**, *80*, 109–117, doi:10.1007/s00280-017-3340-7.
36. Anderson, P.L.; Lamba, J.; Aquilante, C.L.; Schuetz, E.; Fletcher, C.V. Pharmacogenetic characteristics of indinavir, zidovudine, and lamivudine therapy in HIV-infected adults: a pilot study. *J. Acquir. Immune Defic. Syndr. 1999* **2006**, *42*, 441–449, doi:10.1097/01.qai.0000225013.53568.69.
37. Rungtivasuwan, K.; Avihingsanon, A.; Thammajaruk, N.; Mitruk, S.; Burger, D.M.; Ruxrungtham, K.; Punyawudho, B.; Pengsuparp, T. Influence of ABCC2 and ABCC4 Polymorphisms on Tenofovir Plasma Concentrations in Thai HIV-Infected Patients. *Antimicrob. Agents Chemother.* **2015**, *59*, 3240–3245, doi:10.1128/AAC.04930-14.
38. Ban, H.; Andoh, A.; Imaeda, H.; Kobori, A.; Bamba, S.; Tsujikawa, T.; Sasaki, M.; Saito, Y.; Fujiyama, Y. The multidrug-resistance protein 4 polymorphism is a new factor accounting for thiopurine sensitivity in Japanese patients with inflammatory bowel disease. *J. Gastroenterol.* **2010**, *45*, 1014–1021, doi:10.1007/s00535-010-0248-y.
39. den Hoed, M.A.H.; Lopez-Lopez, E.; te Winkel, M.L.; Tissing, W.; de Rooij, J.D.E.; Gutierrez-Camino, A.; Garcia-Orad, A.; den Boer, E.; Pieters, R.; Pluijm, S.M.F.; et al. Genetic and metabolic determinants of methotrexate-induced mucositis in pediatric acute lymphoblastic leukemia. *Pharmacogenomics J.* **2015**, *15*, 248–254, doi:10.1038/tpj.2014.63.
40. Lopez-Lopez, E.; Ballesteros, J.; Piñan, M.; Toledo, J.S. de; Andoin, N.G. de; Garcia-Miguel, P.; Navajas, A.; Garcia-Orad, A. Polymorphisms in the methotrexate transport pathway: a new tool for MTX plasma level prediction in pediatric acute lymphoblastic leukemia. *Pharmacogenet. Genomics* **2013**, *23*, 53–61, doi:10.1097/FPC.0b013e32835c3b24.
41. Angelini, S.; Pantaleo, M.A.; Ravegnini, G.; Zenesini, C.; Cavrini, G.; Nannini, M.; Fumagalli, E.; Palassini, E.; Saponara, M.; Di Battista, M.; et al. Polymorphisms in OCTN1 and OCTN2 transporters genes are associated with prolonged time to progression in unresectable gastrointestinal stromal tumours treated with imatinib therapy. *Pharmacol. Res.* **2013**, *68*, 1–6, doi:10.1016/j.phrs.2012.10.015.
42. Low, S.-K.; Kiyotani, K.; Mushiroda, T.; Daigo, Y.; Nakamura, Y.; Zembutsu, H. Association study of genetic polymorphism in ABCC4 with cyclophosphamide-induced adverse drug reactions in breast cancer patients. *J. Hum. Genet.* **2009**, *54*, 564–571, doi:10.1038/jhg.2009.79.
43. Poonkuzhali, B.; Lamba, J.; Strom, S.; Sparreboom, A.; Thummel, K.; Watkins, P.; Schuetz, E. Association of Breast Cancer Resistance Protein/ABCG2 Phenotypes and Novel Promoter and Intron 1 Single Nucleotide Polymorphisms. *Drug Metab. Dispos.* **2008**, *36*, 780–795, doi:10.1124/dmd.107.018366.
44. Mizuarai, S.; Aozasa, N.; Kotani, H. Single nucleotide polymorphisms result in impaired membrane localization and reduced atpase activity in multidrug transporter ABCG2. *Int. J. Cancer* **2004**, *109*, 238–246, doi:10.1002/ijc.11669.
45. Verboom, M.C.; Kloth, J.S.L.; Swen, J.J.; Sleijfer, S.; Reyners, A.K.L.; Steeghs, N.; Mathijssen, R.H.J.; Gelderblom, H.; Guchelaar, H.-J. Genetic polymorphisms in ABCG2 and CYP1A2 are associated with imatinib dose reduction in patients treated for gastrointestinal stromal tumors. *Pharmacogenomics J.* **2019**, *19*, 473–479, doi:10.1038/s41397-019-0079-z.
46. Han, J.-Y.; Lim, H.-S.; Park, Y.H.; Lee, S.Y.; Lee, J.S. Integrated pharmacogenetic prediction of irinotecan pharmacokinetics and toxicity in patients with advanced non-small cell lung cancer. *Lung Cancer* **2009**, *63*, 115–120, doi:10.1016/j.lungcan.2007.12.003.
47. Hira, D.; Terada, T. BCRP/ABCG2 and high-alert medications: Biochemical, pharmacokinetic, pharmacogenetic, and clinical implications. *Biochem. Pharmacol.* **2018**, *147*, 201–210, doi:10.1016/j.bcp.2017.10.004.
48. De Mattia, E.; Toffoli, G.; Polesel, J.; D'Andrea, M.; Corona, G.; Zagonel, V.; Buonadonna, A.; Dreussi, E.; Cecchin, E. Pharmacogenetics of ABC and SLC transporters in metastatic colorectal cancer patients

receiving first-line FOLFIRI treatment. *Pharmacogenet. Genomics* **2013**, *23*, 549–557, doi:10.1097/FPC.0b013e328364b6cf.

49. Steward, D.J.; Haining, R.L.; Henne, K.R.; Davis, G.; Rushmore, T.H.; Trager, W.F.; Rettie, A.E. Genetic association between sensitivity to warfarin and expression of CYP2C9*3. *Pharmacogenetics* **1997**, *7*, 361–367, doi:10.1097/00008571-199710000-00004.
50. King, B.P.; Khan, T.I.; Aithal, G.P.; Kamali, F.; Daly, A.K. Upstream and coding region CYP2C9 polymorphisms: correlation with warfarin dose and metabolism. *Pharmacogenetics* **2004**, *14*, 813–822, doi:10.1097/00008571-200412000-00004.
51. Sim, S.; Risinger, C.; Dahl, M.; Aklillu, E.; Christensen, M.; Bertilsson, L.; Ingelmansundberg, M. A common novel CYP2C19 gene variant causes ultrarapid drug metabolism relevant for the drug response to proton pump inhibitors and antidepressants. *Clin. Pharmacol. Ther.* **2006**, *79*, 103–113, doi:10.1016/j.clpt.2005.10.002.
52. Chaudhry, A.S.; Prasad, B.; Shirasaka, Y.; Fohner, A.; Finkelstein, D.; Fan, Y.; Wang, S.; Wu, G.; Aklillu, E.; Sim, S.C.; et al. The CYP2C19 Intron 2 Branch Point SNP is the Ancestral Polymorphism Contributing to the Poor Metabolizer Phenotype in Livers with CYP2C19*35 and CYP2C19*2 Alleles. *Drug Metab. Dispos. Biol. Fate Chem.* **2015**, *43*, 1226–1235, doi:10.1124/dmd.115.064428.
53. Zhang, L.; Sarangi, V.; Moon, I.; Yu, J.; Liu, D.; Devarajan, S.; Reid, J.M.; Kalari, K.R.; Wang, L.; Weinshilboum, R. CYP2C9 and CYP2C19: Deep Mutational Scanning and Functional Characterization of Genomic Missense Variants. *Clin. Transl. Sci.* **2020**, *13*, 727–742, doi:10.1111/cts.12758.
54. Morais, S.M. de; Wilkinson, G.R.; Blaisdell, J.; Nakamura, K.; Meyer, U.A.; Goldstein, J.A. The major genetic defect responsible for the polymorphism of S-mephenytoin metabolism in humans. *J. Biol. Chem.* **1994**, *269*, 15419–15422, doi:n/a.
55. Johansson, I.; Lundqvist, E.; Bertilsson, L.; Dahl, M.L.; Sjoqvist, F.; Ingelman-Sundberg, M. Inherited amplification of an active gene in the cytochrome P450 CYP2D locus as a cause of ultrarapid metabolism of debrisoquine. *Proc. Natl. Acad. Sci.* **1993**, *90*, 11825–11829, doi:10.1073/pnas.90.24.11825.
56. Yang, X.; Zhang, B.; Molony, C.; Chudin, E.; Hao, K.; Zhu, J.; Gaedigk, A.; Suver, C.; Zhong, H.; Leeder, J.S.; et al. Systematic genetic and genomic analysis of cytochrome P450 enzyme activities in human liver. *Genome Res.* **2010**, *20*, 1020–1036, doi:10.1101/gr.103341.109.
57. Hu, X.; Zhang, M.; Bai, H.; Wu, L.; Chen, Y.; Ding, L.; Zhao, Z.; Peng, W.; Liu, T.; Song, J.; et al. Antituberculosis Drug-Induced Adverse Events in the Liver, Kidneys, and Blood: Clinical Profiles and Pharmacogenetic Predictors. *Clin. Pharmacol. Ther.* **2018**, *104*, 326–334, doi:10.1002/cpt.924.
58. Kubo, M.; Koue, T.; Maune, H.; Fukuda, T.; Azuma, J. Pharmacokinetics of Aripiprazole, a New Antipsychotic, following Oral Dosing in Healthy Adult Japanese Volunteers: Influence of CYP2D6 Polymorphism. *Drug Metab. Pharmacokinet.* **2007**, *22*, 358–366, doi:10.2133/dmpk.22.358.
59. Roco, Á.; Quiñones, L.; Agúndez, J.A.G.; García-Martín, E.; Squicciarini, V.; Miranda, C.; Garay, J.; Farfán, N.; Saavedra, I.; Cáceres, D.; et al. Frequencies of 23 Functionally Significant Variant Alleles Related with Metabolism of Antineoplastic Drugs in the Chilean Population: Comparison with Caucasian and Asian Populations. *Front. Genet.* **2012**, *3*, doi:10.3389/fgene.2012.00229.
60. Saito, T.; Gutiérrez Rico, E.M.; Kikuchi, A.; Kaneko, A.; Kumondai, M.; Akai, F.; Saigusa, D.; Oda, A.; Hirasawa, N.; Hiratsuka, M. Functional characterization of 50 CYP2D6 allelic variants by assessing primaquine 5-hydroxylation. *Drug Metab. Pharmacokinet.* **2018**, *33*, 250–257, doi:10.1016/j.dmpk.2018.08.004.
61. Leathart, J.B.; London, S.J.; Steward, A.; Adams, J.D.; Idle, J.R.; Daly, A.K. CYP2D6 phenotype-genotype relationships in African-Americans and Caucasians in Los Angeles. *Pharmacogenetics* **1998**, *8*, 529–541, doi:10.1097/00008571-199812000-00010.
62. Iacobucci, I.; Lonetti, A.; Candoni, A.; Sazzini, M.; Papayannidis, C.; Formica, S.; Ottaviani, E.; Ferrari, A.; Michelutti, A.; Simeone, E.; et al. Profiling of drug-metabolizing enzymes/transporters in CD33+ acute myeloid leukemia patients treated with Gemtuzumab-Ozogamicin and Fludarabine, Cytarabine and Idarubicin. *Pharmacogenomics J.* **2013**, *13*, 335–341, doi:10.1038/tpj.2012.13.
63. Fairbrother, K.S.; Grove, J.; de Waziers, I.; Steimel, D.T.; Day, C.P.; Crespi, C.L.; Daly, A.K. Detection and characterization of novel polymorphisms in the CYP2E1 gene. *Pharmacogenetics* **1998**, *8*, 543–552, doi:10.1097/00008571-199812000-00011.
64. Werk, A.N.; Cascorbi, I. Functional Gene Variants of CYP3A4. *Clin. Pharmacol. Ther.* **2014**, *96*, 340–348, doi:10.1038/clpt.2014.129.

65. Żochowska, D.; Wyzgał, J.; Pączek, L. Impact of CYP3A4*1B and CYP3A5*3 polymorphisms on the pharmacokinetics of cyclosporine and sirolimus in renal transplant recipients. *Ann. Transplant.* **2012**, *17*, 36–44, doi:10.12659/aot.883456.
66. Wang, D.; Sadee, W. CYP3A4 intronic SNP rs35599367 (CYP3A4*22) alters RNA splicing. *Pharmacogenet. Genomics* **2016**, *26*, 40–43, doi:10.1097/FPC.0000000000000183.
67. Wang, D.; Guo, Y.; Wrighton, S.A.; Cooke, G.E.; Sadee, W. Intronic polymorphism in CYP3A4 affects hepatic expression and response to statin drugs. *Pharmacogenomics J.* **2011**, *11*, 274–286, doi:10.1038/tpj.2010.28.
68. Kuehl, P.; Zhang, J.; Lin, Y.; Lamba, J.; Assem, M.; Schuetz, J.; Watkins, P.B.; Daly, A.; Wrighton, S.A.; Hall, S.D.; et al. Sequence diversity in CYP3A promoters and characterization of the genetic basis of polymorphic CYP3A5 expression. *Nat. Genet.* **2001**, *27*, 383–391, doi:10.1038/86882.
69. Birdwell, K.; Decker, B.; Barbarino, J.; Peterson, J.; Stein, C.; Sadee, W.; Wang, D.; Vinks, A.; He, Y.; Swen, J.; et al. Clinical Pharmacogenetics Implementation Consortium (CPIC) Guidelines for *CYP3A5* Genotype and Tacrolimus Dosing. *Clin. Pharmacol. Ther.* **2015**, *98*, 19–24, doi:10.1002/cpt.113.
70. Court, M.H.; Peter, I.; Hazarika, S.; Vasiadi, M.; Greenblatt, D.J.; Lee, W.M.; The Acute Liver Failure Study Group Candidate Gene Polymorphisms in Patients with Acetaminophen-Induced Acute Liver Failure. *Drug Metab. Dispos.* **2014**, *42*, 28–32, doi:10.1124/dmd.113.053546.
